# Modulation of Hyperpolarization-Activated Inward Current and Thalamic Activity Modes by Different Cyclic Nucleotides

**DOI:** 10.3389/fncel.2018.00369

**Published:** 2018-10-24

**Authors:** Maia Datunashvili, Rahul Chaudhary, Mehrnoush Zobeiri, Annika Lüttjohann, Evanthia Mergia, Arnd Baumann, Sabine Balfanz, Björn Budde, Gilles van Luijtelaar, Hans-Christian Pape, Doris Koesling, Thomas Budde

**Affiliations:** ^1^Institut für Physiologie I, Westfälische Wilhelms-Universität, Münster, Germany; ^2^Institut für Pharmakologie und Toxikologie, Ruhr-Universität Bochum, Bochum, Germany; ^3^Institute of Complex Systems, Forschungszentrum Jülich, Jülich, Germany; ^4^Donders Centre for Cognition, Radboud University, Nijmegen, Netherlands

**Keywords:** thalamus, dLGN, cyclic nucleotides, NO-GC2, HCN channels, I_h_ current, I_KIR_ current, slow oscillations

## Abstract

The hyperpolarization-activated inward current, I_h_, plays a key role in the generation of rhythmic activities in thalamocortical (TC) relay neurons. Cyclic nucleotides, like 3′,5′-cyclic adenosine monophosphate (cAMP), facilitate voltage-dependent activation of hyperpolarization-activated cyclic nucleotide-gated (HCN) channels by shifting the activation curve of I_h_ to more positive values and thereby terminating the rhythmic burst activity. The role of 3′,5′-cyclic guanosine monophosphate (cGMP) in modulation of I_h_ is not well understood. To determine the possible role of the nitric oxide (NO)-sensitive cGMP-forming guanylyl cyclase 2 (NO-GC2) in controlling the thalamic I_h_, the voltage-dependency and cGMP/cAMP-sensitivity of I_h_ was analyzed in TC neurons of the dorsal part of the lateral geniculate nucleus (dLGN) in wild type (WT) and NO-GC2-deficit (NO-GC2^−/−^) mice. Whole cell voltage clamp recordings in brain slices revealed a more hyperpolarized half maximal activation (V_1/2_) of I_h_ in NO-GC2^−/−^ TC neurons compared to WT. Different concentrations of 8-Br-cAMP/8-Br-cGMP induced dose-dependent positive shifts of V_1/2_ in both strains. Treatment of WT slices with lyase enzyme (adenylyl and guanylyl cyclases) inhibitors (SQ22536 and ODQ) resulted in further hyperpolarized V_1/2_. Under current clamp conditions NO-GC2^−/−^ neurons exhibited a reduction in the I_h_-dependent voltage sag and reduced action potential firing with hyperpolarizing and depolarizing current steps, respectively. Intrathalamic rhythmic bursting activity in brain slices and in a simplified mathematical model of the thalamic network was reduced in the absence of NO-GC2. In freely behaving NO-GC2^−/−^ mice, delta and theta band activity was enhanced during active wakefulness (AW) as well as rapid eye movement (REM) sleep in cortical local field potential (LFP) in comparison to WT. These findings indicate that cGMP facilitates I_h_ activation and contributes to a tonic activity in TC neurons. On the network level basal cGMP production supports fast rhythmic activity in the cortex.

## Introduction

Cyclic nucleotides, like cyclic adenosine monophosphate (cAMP) and cyclic guanosine monophosphate (cGMP) bind to the hyperpolarization-activated cyclic nucleotide-gated (HCN) channels and stabilize their open state (Zagotta et al., 2003). HCN channels represent the molecular basis of the hyperpolarization-activated current, termed I_h_ (Pape, 1996). HCN isoforms (HCN1-4) reveal different characteristics with respect to voltage dependency, activation kinetics and cyclic nucleotide sensitivity (He et al., 2014). HCN2 and the HCN4 isoforms (Ludwig et al., 2003; Notomi and Shigemoto, 2004) are strongly modulated by cAMP in thalamocortical (TC) relay neurons (Kanyshkova et al., 2009, 2012). A number of brain rhythms are controlled by HCN channels, and epileptogenesis in the TC system is accompanied by changes in HCN expression levels and altered properties of I_h_, including cAMP—sensitivity (Budde et al., 2005; Kanyshkova et al., 2012). In thalamic neurons of the rodent brain, I_h_ contributes to the resting membrane potential (RMP) and determines cell type-specific firing patterns and postnatal changes in HCN isoform expression profiles are accompanied by the maturation of sleep-related slow oscillations (Meuth et al., 2006; Kanyshkova et al., 2009).

In the dorsal lateral geniculate nucleus (dLGN), neuronal nitric oxide synthase (nNOS) was found in interneurons and cholinergic afferents arising from the ascending brainstem system (Gabbott and Bacon, 1994). NO exerts an important role in behavioral state-dependent gating of visual information and regulating TC oscillations (Pape and Mager, 1992; Yang and Cox, 2008). Thalamic NO concentrations increase during wakefulness and rapid eye movement (REM) sleep and decrease during slow wave sleep (SWS; Burlet and Cespuglio, 1997), pointing to a possible role of NO in regulation of arousal and REM sleep.

NO has been identified as an important modulator of HCN channel activity mediated by the NO-sensitive soluble NO-GC1 and NO-GC2 (Russwurm et al., 2013). Although cyclic nucleotide-dependent modulation of I_h_ in the thalamus under physiological and pathophysiological conditions has been assessed before (Pape, 1996; He et al., 2014), it is not known whether I_h_ in TC neurons is under the simultaneous control of both cAMP and cGMP. To address this issue, we studied I_h_ properties in wild type (WT) and NO-GC2-deficient mice, in the presence of adenylyl and guanylyl cyclase inhibitors as well as by intracellular application of cyclic nucleotides. By combining *in vitro* voltage and current clamp methods, we examined the characteristics of I_h_ current as well as the passive and active properties of NO-GC2^−/−^ TC cells. By means of *in vitro* and *in vivo* field potential recordings we studied intrathalamic and cortical activities. Based on these results the present study provides a detailed description of the role of cGMP in the regulation of intrathalamic and cortical activities.

## Materials and Methods

### Preparation of Coronal dLGN Slices

All animal work has been approved by local authorities (review board institution: Landesamt für Natur, Umwelt und Verbraucherschutz Nordrhein-Westfalen; approval ID: 84-02.04.2015.A574, 84-02.05.50.15.026). Experiments were performed on NO-GC2-deficient mice (Mergia et al., 2006) ranging in age from postnatal day P16 to P35. These mice lack the α2 subunit of NO-dependent soluble guanylyl cyclase while the α1 and β1 subunits can assemble to enzymatically active NO-GC1. NO-GC2^−/−^ mice were produced by breeding heterozygous mice or homozygous mice of the F1 generation. Genotyping of the mice was performed by PCR analysis of DNA extracted from ear biopsies. As the knockout strain was backcrossed over 10 generations onto C57BL/6J background, C57BL/6J mice (postnatal day P16 to P35) were used as WT controls (WT). Mice were anesthetized with isoflurane (3.5 vol%) and sacrificed. After surgically removing their skull cap caudal to bregma, a block of brain tissue containing the thalamus was removed from the cranial vault and submerged in ice-cold aerated (O_2_) saline containing (in mM): sucrose, 200; PIPES, 20; KCl, 2.5; NaH_2_PO_4_, 1.25; MgSO_4_, 10; CaCl_2_, 0.5; dextrose, 10; pH 7.35, with NaOH. Thalamic slices (250–300 μm thick) were prepared as coronal sections on a vibratome. Slices were transferred to a holding chamber and kept submerged (at 30°C for 30 min, thereafter at room temperature) in artificial cerebrospinal fluid (ACSF) containing (in mM): NaCl, 125; KCl, 2.5; NaH_2_PO_4_, 1.25; NaHCO_3_, 24; MgSO_4_, 2; CaCl_2_, 2; dextrose, 10; pH adjusted to 7.35 by bubbling with carbogen (95% O_2_ and 5% CO_2_ gas mixture).

### Voltage Clamp Recordings

Recordings were done on visually identified TC neurons of the dLGN in a solution containing (in mM): NaCl, 120; KCl, 2.5; NaH_2_PO_4_, 1.25; HEPES, 30; MgSO_4_, 2; CaCl_2_, 2; dextrose, 10; pH 7.35 adjusted with HCl. For some recordings, bicarbonate (NaHCO_3_) buffered ACSF was used (in mM): NaCl, 125; KCl, 2.5; NaH_2_PO_4_, 1.25; NaHCO_3_, 24; MgSO_4_, 2; CaCl_2_, 2; dextrose, 10; pH adjusted to 7.35 by bubbling with carbogen. In order to block inward rectifying K^+^ and K_2P_ channels, 0.5 mM BaCl_2_ was added to the solution. Whole-cell recordings were made from the soma of TC neurons at 30–32°C. Membrane currents were measured with glass microelectrodes pulled from borosilicate glass capillaries (GC150T-10; Clark Electromedical Instruments, Pangbourne, UK) filled with (in mM): K-gluconate, 95; K_3_-citrate, 20; NaCl, 10; HEPES, 10; MgCl_2_, 1; CaCl_2_, 0.5; BAPTA, 3; Mg-ATP, 3; Na_2_-GTP, 0.5. The internal solution was set to a pH of 7.25 with KOH and an osmolality of 295 mOsm/kg. A 0.2 μm pore size sterile filter (MP, Hennigsdorf, Germany) was placed between the needle and the syringe to fill the electrodes. Patch electrodes were connected to an EPC-10 amplifier (HEKA Elektronik, Lamprecht, Germany) via a chlorinated silver wire. The resistances of electrodes were in the range of 2.5–3.5 MΩ. Access resistances were between 8 MΩ and 20 MΩ. Series resistance compensation of >50% was routinely applied. Recordings started 2–3 min after obtaining the whole-cell configuration. Voltage clamp experiments were controlled by the software Pulse or PatchMaster (HEKA Elektronik) operating on an IBM-compatible personal computer. All recordings were corrected offline for a liquid junction potential of 10 mV (V_M_ = V_P_ – 10 mV; with V_M_ = membrane potential and V_P_ = pipette voltage). Care was exercised to monitor series resistance and recordings were terminated whenever a significant increase (>20%) occurred.

The voltage protocol used to examine I_h_ (Kanyshkova et al., 2012) was designed in order to increase the stability of whole cell recordings and account for increasingly fast activation kinetics of the current. Therefore the pulse length was shortened by 500 ms with increasing hyperpolarization (3.5 s pulse length at −130 mV). Steady-state activation of I_h_, p(V), was estimated by normalizing the mean tail current amplitudes (I) 50–100 ms after stepping to a constant potential from a variable amplitude step using the following function (equation 1):

(1) $$\text{P}(\text{V})=(\text{I}-{\text{I}}_{\text{min}})/({\text{I}}_{\text{max}}-{\text{I}}_{\text{min}})$$

with I_max_ being the tail current amplitude for the voltage step from −130 mV to −100 mV and I_min_ for the voltage step from −40 mV to −100 mV, respectively. I_h_ activation was well accounted for by a Boltzmann function of the following form (equation 2):

(2) $$\text{p}(\text{V})=1/(1+\text{e}\text{x}\text{p}((\text{V}-{\text{V}}_{1/2})/\text{k}))$$

where V_1/2_ is the voltage of half-maximal activation and k the slope factor.

The current density was calculated by dividing the I_h_ amplitude at −130 mV (i.e., subtracting the instantaneous current amplitude from the steady-state current) by the membrane capacitance obtained during whole cell recordings.

The time course of I_h_ activation in TC neurons at a temperature of 30–32°C was best approximated by the following double-exponential equation:

(3) $$I_{\text{h}}(t)=A_{1}(1-\exp^{-t/\tau \text{fast}})+A_{2}(1-\exp^{-t/\tau \text{slow}}),$$

Where I_h_(*t*) is the time (ms), *A*_1_ and *A*_2_ are current amplitudes (pA), and τ_fast_ and τ_slow_ are time constants (ms), respectively. Currents evoked by voltage steps to −130 mV were analyzed.

A series of hyperpolarizing (500 ms) voltage steps in −10 mV increments were injected from the holding potential of −60 to −130 mV in order to evoke inwardly rectifying potassium (I_KIR_) current. I_KIR_ currents were isolated from I_h_ by applying 20 μM ZD7288. I_KIR_ amplitudes were measured manually as the difference of the peak and the steady state current at the beginning and at the end of voltage pulses, respectively.

### Current Clamp Recordings and Determination of the Intrinsic Electrophysiological Properties

The active and passive membrane properties of TC neurons were determined in current clamp mode. Recordings were performed at RMP in Ba^2+^-free extracellular solution containing (in mM): NaCl, 125; KCl, 2.5; NaH_2_PO_4_, 1.25; NaHCO_3_, 24; MgSO_4_, 2; CaCl_2_, 2; dextrose, 10; pH adjusted to 7.35 by bubbling with carbogen. In order to compare the effects of different buffers and Ba^2+^ ions on intrinsic membrane properties of TC neurons, 0.5 mM BaCl_2_ was added in HEPES and NaHCO_3_ buffered extracellular solutions. Analysis was performed according to established procedures (Leist et al., 2016). Only cells with overshooting APs were included for analysis. The stimulation protocol contained hyperpolarizing and depolarizing current steps (1 s duration, from −230 pA to +370 pA with 40 pA increments; for 8-Br-cGMP experiments, a protocol with steps of 1 s duration, from −120 pA to +260 pA with 20 pA increments, was used). Membrane input resistance (R_in_) was deduced from the slope of the current-voltage (I-V) relationship obtained from current injections of −30 and 50 pA. Membrane time constants (τ_m_) were obtained by fitting single or double exponentials (FitMaster, HEKA Elektronik) to negative voltage deflections induced by hyperpolarizing current injections of −30 pA. The I_h_-dependent anomalous rectification (or voltage sag) of current injection of −230 pA was calculated as the change between the maximal and steady state voltage deflection (at the end of hyperpolarizing current injection). APs were detected manually by setting an amplitude threshold (V_thresh_). FitMaster (HEKA Elektronik) and Clampfit 10.7 (Axon Molecular Devices, Sunnyvale, CA, USA) software was used for the analyses.

### Immunofluorescence

WT mice were transcardially perfused with 4% (w/v) phosphate-buffered paraformaldehyde (PFA). Brains were removed and post fixed overnight in 4% PFA and later in 30% (w/v) sucrose for 48–72 h. Free-floating coronal sections (40 μm) were cut and slices were collected in phosphate-buffered saline (PBS). Sections were rinsed three times for 10 min in PBS. Slices were then incubated for 2 h in PBS supplemented with 10% (v/v) normal goat serum, Triton-X100 (0.3% (w/v)), and 3% (w/v) bovine serum albumin (BSA). Finally sections were incubated with primary antibodies overnight at 4°C. Polyclonal rabbit (rb) anti-NO-GCα_2_ (1:1,1000; ab42108, Abcam, USA) antibody was utilized to detect a localization of dLGN. Several studies using this antibody revealed detecting of a single protein band of the correct size in quantitative Western blot experiments, thereby pointing to specificity of the reaction (Backer et al., 2008; Thoonen et al., 2015; product data sheet Abcam). In a similar way the monoclonal mouse anti-postsynaptic density protein 95 (PSD95; 1:1,000; 10011435, Cayman, USA) antibody which marks the postsynaptic membrane revealed a single protein band in Wetsern blots (Yao et al., 2004). After incubation with primary antibodies, sections were washed three times for 10 min in PBS and then transferred to the secondary antibody solution (Alexa Fluor 488 goat anti-rabbit-IgG, 1:1,000 and Alexa Fluor 568 goat anti-mouse-IgG, 1:1,000) for 2 h. Finally, sections were washed three times for 10 min in PBS and mounted with a mounting medium (VECTASHIELD, Vector Laboratories Inc., Burlingame, CA, USA) for confocal microscopy (Nikon eC1plus) equipped with a CFI75 LWD × 16/0.8 NA objective (Nikon). Omission of primary or secondary antibodies from the staining procedure resulted in a lack of fluorescent signals.

### Quantification of cGMP in Tissue Samples

WT mice of different postnatal development age (P7, P21 and P105) were sacrificed and coronal slices were prepared on a vibratome. Separate tissues containing dLGN, ventrobasal thalamic complex (VB) and somatosensory cortex (SSC) were placed in ice-cold buffer containing (in mM): sucrose, 200; PIPES, 20; KCl, 2.5; NaH_2_PO_4_, 1.25; MgSO_4_, 10; CaCl_2_, 0.5; dextrose, 10; pH 7.35 with NaOH and further incubated in an oxygenated salt solution (in mM: NaCl, 125; KCl, 2.5; NaH_2_PO_4_, 1.25; HEPES, 30; glucose, 10; CaCl_2_, 2; MgCl_2_, 1; IBMX, 0.05; pH 7.3) for 2 h. Samples were mechanically homogenized at room temperature. To eliminate any non-solubilized material, samples were centrifuged at 1,000× *g* for 10 min at 4°C. The supernatant was transferred to a new Eppendorf cup and kept on ice, snap frozen in liquid nitrogen and stored at −80°C before further use.

Prior to cGMP quantification, samples were thawed on ice and 50 μl was used for Bradford protein assay. A cyclic GMP ELISA Kit (Prod. No. 581021, Cayman Chemicals, Ann Arbor, MI, USA) was used to quantify cGMP. Briefly, all samples were treated with trichloroacetic acid (TCA, final concentration 5% (w/v)) to precipitate proteins. After centrifugation, the supernatant was extracted with ether to remove any TCA residuals. An acetylation step for tissue samples was performed according to the manufacturer’s protocol. The final assay set up, luminescent measurement and analysis was performed as suggested by the manufacturer. We used a Fluostar Omega fluorescence reader (BMG Labtech, Ortenberg, Germany) for data acquisition and Microsoft Excel 2011 version 14.0 to analyze the data.

### Rhythmic Burst Activity Recordings in Thalamic Slices

Horizontal brain slices were transferred to an interface chamber and recordings were performed at 32 ± 1°C. The superfusion solution consisted of (in mM): NaCl, 125; KCl, 2.5; NaHCO_3_, 26; NaH_2_PO_4_, 1.25; MgCl_2_, 1; CaCl_2,_ 2; glucose, 10; pH 7.35 adjusted with carbogen. Rhythmic burst activity was induced through stimulation (1 ms, 1.45 mA) of the internal capsule (IC) using a pair of tungsten electrodes (with 50–100 MΩ resistance). Stimulation electrodes were connected to custom-made amplifier and stimulus isolator, and duration of stimulus was controlled by WinLTPd101 software (WinLTP Ltd, University of Bristol, UK). Network activity was measured in VB using a glass electrode (GC150T-10; Clark Electromedical Instruments, Harvard, UK) with a resistance of 0.5–2 MΩ. Burst firing was characterized by at least three high-frequency spikes with an intra-burst frequency interval of >100 Hz and inter-burst interval not more than 500 ms. Activity was analyzed in a time interval ranging from 50 ms to 100 ms up to 2–3 s after stimulation of the IC. Analysis was performed offline using Clampfit 10.7 and Peak v1.0 software.

### Electrode Implantation and LFP Recordings for *in vivo* Electrophysiology

Mature properties of I_h_ were reached in young mice postnatal between P20 and P30. Similar mature sleep pattern were found in young (P18) and adult (P90) rodents based on the analyses of cortical electroencephalogram (EEG) recordings, in which well-developed high amplitude delta waves distinguished NREM sleep from wakefulness. 3 to 5 months old adult male NO-GC2^−/−^ and WT mice were used for the *in vivo* experiments. Before surgery each animal was kept individually for 1 week in 12 h light/dark conditions (6 a.m.–6 p.m. light on period) and had unrestricted access to water and food. Implantation of the local field potential (LFP) recording electrodes was performed in a stereotactic frame (David Kopf Instruments, Tujunga, CA, USA) in animals under pentobarbital anesthesia (50 mg kg^−1^ i.p.) supplemented by a subcutaneous injection of carprofen (rimadyl; 5 mg/kg^−1^). Holes were drilled into the skull of the right hemisphere for inserting the silver recording electrode within the somatosensory cortex (SSC) (A/P = 0, M/L = 3, depth = −1.2; referenced to Bregma according to the mouse brain atlas), as well as for the reference and ground electrodes, which were placed over the cerebellum. The electrode assembly was fixed to the skull using dental acrylic cement (Pulpdent Glasslute, Watertown, MA, USA). After 1 week of recovery period the mice were habituated to the recording chamber. The LFP signal of each mouse was recorded for 2 h between 6 and 8 a.m. corresponding to the first 2 h of the light period. The LFP signal was amplified with a physiological amplifier (DPA-2F, Science Products, Hofheim, Germany), filtered by a band pass filter with cut-off points at 1 (HP) and 30 (LP) Hz and digitalized with a constant sampling rate of 2 kHz by multichannel continuous recording system (CED1401, Cambridge Electronic Design, Cambridge, England). In parallel, behavioral activity of mice was registered using a Passive Infrared Recording System (PIR, RK2000DPC LuNAR PR Ceiling Mount, Rokonet RISCO Group S.A., Drogenbos, Belgium). Following LFP recordings, animals were deeply anesthetized with an overdose of pentobarbital (i.p. injection) and the brains were removed for histological verification of the correct electrode positions.

### Analysis of LFP Activity

LFPs were inspected offline by trained electrophysiologist (blinded for genotype) using Spike2 analysis software (Version 7.08, Cambridge Electronic Design, Germany). Recordings were subjected to Fast Fourier Transformation (FFT) in the 1–30 Hz range. Twenty epochs of 10 s duration were chosen from four behavioral states for power spectral density (PSD) analyses: active wakefulness (AW), REM sleep, light SWS (LSWS) and deep SWS (DSWS). For all epochs the EEG power in the delta (*δ* = 1–4 Hz), theta (*θ* = 5–8 Hz), alpha (*α* = 9–12 Hz) and beta (*β* = 13–30 Hz) frequency ranges were calculated. Epochs were scored visually according to the following criteria: AW was characterized by high activity on the PIR and LFP which was dominated by a mixture of theta activity with higher frequencies, the amplitude was generally low. Low frequencies with intermediate amplitude and spindle activity and a low PIR were prevalent during LSWS. During DSWS, the LFP was dominated by low frequency, high amplitude delta activity and a low PIR as well. REM sleep was characterized by high frequency and low amplitude EEG activity, with predominantly theta activity. During REM sleep no movements were detected by PIR, indicating that the animal was immobile except for facial and bodily twitches. For each behavioral state the spectral power of the LFP epochs were assessed via a time frequency analysis (TFA) using Hanning tapering. Assessment of spectral power was performed in 1 s timeframes shifting along the 10 s epochs in steps of 50 ms. TFA was performed for the frequency range of 1–30 Hz using Fieldtrip software, an open-source Matlab-based toolbox for advanced analysis of electrophysiological data (Oostenveld et al., 2011). From each animal 20 epochs of 10 s duration spectral power was averaged over time for different frequency bands. Both raw and normalized EEG power spectra were used for statistical analysis. Normalized power spectra were used to control individual differences in EEG amplitude and PSD’s. To do so, the total power of 1–30 Hz was set at 100% and percentage for δ, θ, α and β frequencies were calculated.

### Computer Modeling

Simulations were conducted within the NEURON simulation tool (Hines and Carnevale, 2001; Meuth et al., 2005) based on a modified version of an intrathalamic network model consisting of four cells (Destexhe et al., 1998). The four-cell model comprised of two spontaneously pacemaking TC neurons and two reticular thalamic (nRT) neurons interacting via GABA_A_, GABA_A+B_ and AMPA synapses. While nRT neuron parameters were not changed, the I_h_ module of both TC neurons was modified by introducing activation kinetics, V_1/2_, k and conductance values as obtained from our voltage-clamp recordings in 11 control and 13 knockout cells (Kanyshkova et al., 2009, 2012).

### Materials

8-bromo-adenosine 3′,5′-cyclic monophosphate (8-Br-cAMP) sodium salt, 8-bromo-guanosine 3′,5′-cyclic monophosphate (8-Br-cGMP) sodium salt (Tocris, R+D Systems, Wiesbaden, Germany), Cytidine-3′,5′-cyclic monophosphate (cCMP), sodium salt and Uridine-3′,5′-cyclic monophosphate (cUMP), sodium salt (BIOLOG Life Science Institute, Bremen, Germany) were added to the recording pipette. Properties of I_h_ were determined 10–15 min after obtaining the whole-cell configuration. The soluble guanylyl cyclase inhibitor 1H-[1,2,4]Oxadiazolo[4,3-a]quinoxalin-1-one (ODQ; Sigma, Munich, Germany) was prepared as a stock solution in DMSO (10 mM) and diluted in ACSF to obtain the final concentration (10 μM). The concentration of DMSO was below 0.1%. Slices were kept for 10 min in ODQ before starting the recordings. In order to block adenylyl cyclase, the slices were preincubated with 200 μM SQ 22536 (Tocris) for 2 h. HCN channels were blocked by washing the slices for 10 min with 20 μM ZD7288 (Abcam, Cambridge, UK). Inward-rectifier K^+^ channels were blocked by Tertiapin-Q (Tocris).

### Data and Statistical Analysis

All results are presented as mean ± SEM if not mentioned otherwise. By default statistical significance was tested using the nonparametric Mann-Whitney test. For normally distributed data Student’s *t*-test was used (Graph Pad Prism software; Graph Pad, San Diego, CA, USA; OriginLab software, Additive GmbH, Friedrichsdorf, Germany). For multiple comparisons ANOVA testing (Graph Pad Prism) was used. For statistical comparison of NO-GC2^−/−^ and WT mice recorded *in vivo*, data were subjected to a Repeated-Measures-ANOVA with spectral power as dependent variable, mouse strain (NO-GC2^−/−^, WT) between subjects factor and frequency band (delta, theta, alpha, beta) as within subjects factor. This analysis was performed for each of the four behavioral states (AW, REM, LSWS, DSWS) using IBM-SPSS Version 22. Differences were considered statistically significant if *P* < 0.05. *,**,*** indicate *P* < 0.05, *P* < 0.01, *P* < 0.001, respectively.

## Results

### Modulation of I_h_ in TC Neurons by Cyclic Nucleotides

**Figure 1:**
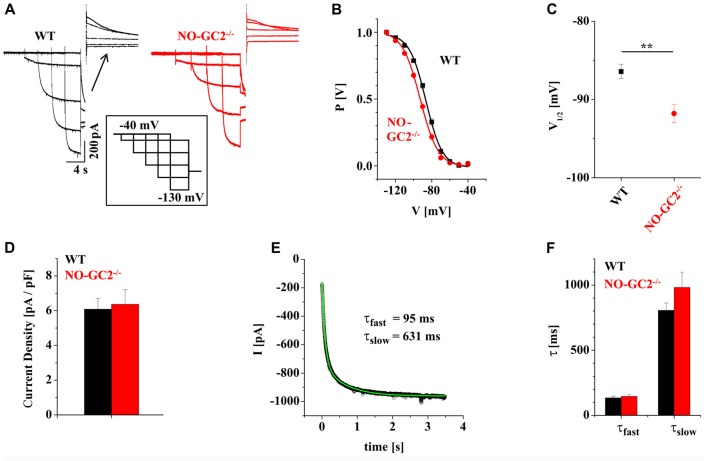
Characterization of I_h_ in NO-GC2^−/−^ mice. **(A)** Example traces of I_h_ recorded from wild type (WT; black) and NO-GC2^−/−^ (red) mice. During whole cell patch clamp recordings holding potential was switched from −40 mV to −130 mV with 10 mV increments before stepping to −100 mV. Tail current amplitudes (shown above the traces) at −100 mV were used to construct activation curves. **(B)** Steady-state activation curves obtained by plotting normalized I_h_ amplitudes vs. voltage and fitting data points with the Boltzmann equation (solid lines). The voltage dependency of I_h_ was shifted to more negative potentials in NO-GC2^−/−^ (red symbols) compared to WT (black symbols) animals. **(C)** Mean values of half maximal activation (V_1/2_) show a more hyperpolarized activation potential of I_h_ in NO-GC2^−/−^ (red symbol) compared to WT (black symbol) mice. **(D)** I_h_ current density in thalamocortical (TC) relay neurons from WT (black bar) and NO-GC2^−/−^ (red bar) mice was unchanged. **(E)** Activation kinetics of I_h_ in TC neurons were well fitted with two exponentials. **(F)** Fast (left bars) and slow (right bars) activation time constants were unchanged in neurons examined from WT (black bars) and NO-GC2^−/−^ (red bars) mice. **P < 0.01.

Cyclic nucleotides, like cAMP and cGMP can influence neuronal signaling via modulation of ion channel activity either by direct binding interaction or indirectly by cyclic nucleotide- dependent phosphorylation of ion channels. Modulation by cAMP is a well characterized property of HCN channels in rodent TC neurons, but the role of cGMP is not fully understood. In the present study, thalamic I_h_ properties were analyzed in WT and NO-GC2^−/−^ mice (Figure 1A). Analyses of activating and deactivating currents revealed that the loss of NO-GC2 was associated with a significant negative shift in voltage-dependency of activation (WT: V_1/2_ = −86.4 ± 0.9 mV, *n* = 11; NO-GC2^−/−^: V_1/2_ = −91.8 ± 1.1 mV, *n* = 13; *P* < 0.05; Figures 1B,C), whereas current density remained unaltered (WT: I_h_ = 6.1 ± 0.6 pA/pF, *n* = 11; NO-GC2^−/−^: I_h_ = 6.4 ± 0.8 pA/pF, *n* = 13; *P* > 0.05; Figure 1D). Activation kinetics were best approximated by a double-exponential function (Figure 1E), and revealed no changes (WT: τ_fast_/τ_slow_ = 135 ± 13 ms/806 ± 60 ms, *n* = 11; NO-GC2^−/−^: τ_fast_/τ_slow_ = 146 ± 15 ms/981 ± 117 ms, *n* = 13; *P*’s > 0.05; Figure 1F).

**Figure 2:**
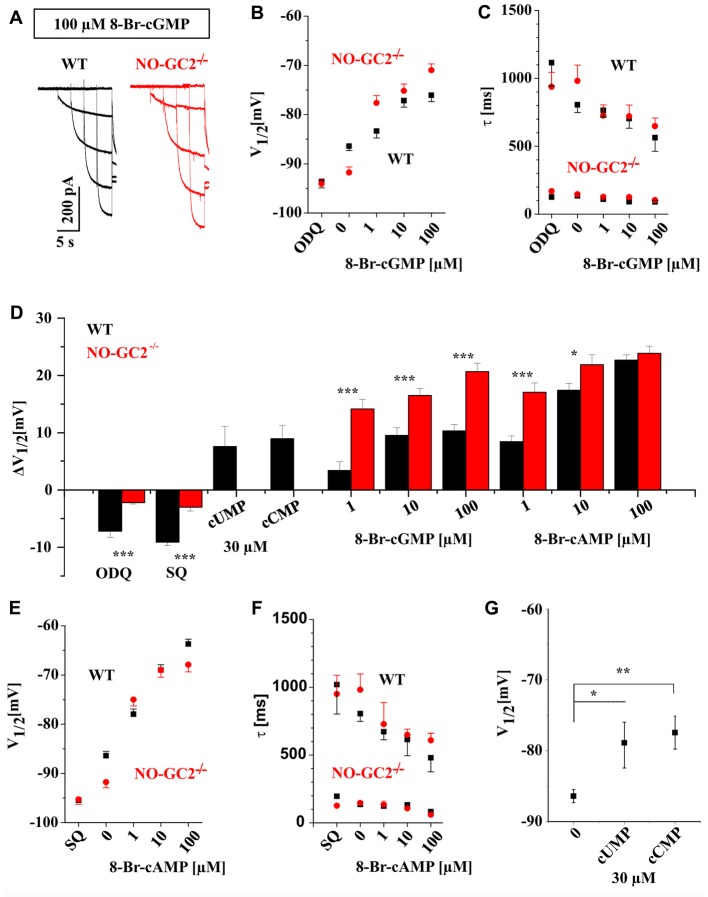
Thalamic I_h_ is modulated by different cyclic nucleotides. **(A)** Example traces of I_h_ recorded from WT (black) and NO-GC2^−/−^ (red) mice in the presence of 100 μM 8-Br-cyclic guanosine monophosphate (cGMP) in the intracellular solution. **(B)** Different concentrations of 8-Br-cGMP induced a dose-dependent depolarizing shift of V_1/2_ in both mouse strains. Stronger effects were found in NO-GC2^−/−^ (red symbols) for some concentrations compared to WT (black symbols). The selective sGC inhibitor ODQ significantly shifted V_1/2_ to more negative potentials in WT but not in NO-GC2^−/−^mice. **(C)** Activation kinetics of hyperpolarization activatedcyclic nucleotide-gated (HCN) channels were enhanced by different concentrations of 8-Br-cGMP in both strains (WT—black symbols; NO-GC2^−/−^—red symbols). The upper and lower panels show the slow and fast time constants of activation. ODQ slowed the slow component of HCN channel kinetics in WT but not in NO-GC2^−/−^ mice. **(D)** Calculation of ΔV_1/2_ revealed different sensitivity of thalamic I_h_ current to cyclic nucleotides and adenylyl-and guanylyl-cyclase inhibitors between WT and NO-GC2^−/−^ mice. **(E)** Effects of various concentrations of 8-Br-cyclic adenosine monophosphate (cAMP) and the adenylyl cyclase inhibitor SQ22536 on V_1/2_ (WT—black symbols; NO-GC2^−/−^ – red symbols). **(F)** Effects of various concentrations of 8-Br-cAMP and the adenylyl cyclase inhibitor SQ22536 on activation time constants of HCN channels (WT—black symbols; NO-GC2^−/−^—red symbols). **(G)** Modulation of V_1/2_ of I_h_ by cCMP and cUMP as indicated. *,**,*** indicate *P* < 0.05, *P* < 0.01, *P* < 0.001, respectively.

We next assessed the effects of cGMP on I_h_ current properties by supplying defined 8-Br-cGMP concentrations (1, 10, 100 μM) via the recording pipette. The experiments revealed dose-dependent alterations of I_h_ in both, WT and NO-GC2^−/−^ animals (Figure 2A). While V_1/2_ was positively shifted (Figure 2B), activation kinetics were enhanced (Figure 2C). For better comparison of different sensitivity between the strains, ΔV_1/2_ was calculated (Figure 2D). TC neurons from NO-GC2^−/−^ revealed increased sensitivity to all applied concentrations of 8-Br-cGMP (ΔV_1/2_: 1 μM = 14.2 ± 1.7 mV, *n* = 7; 10 μM = 16.5 ± 1.2 mV, *n* = 8; 100 μM = 20.7 ± 1.5 mV, *n* = 9), compared to WT (ΔV_1/2_: 1 μM = 3.4 mV, *n* = 8; 10 μM = 9.6 ± 1.3 mV, *n* = 11; 100 μM = 10.3 ± 1.1 mV, *n* = 9; *P*’s < 0.05; Figure 2D). Preincubation of slices in 10 μM ODQ induced a negative shift of V_1/2_ in WT (V_1/2_ = −93.6 ± 1.3 mV; ΔV_1/2_ = −7.2 ± 1.1 mV, *n* = 8), while it did not cause significant changes in GC2 knockout mice (V_1/2_ = −94.1 ± 0.9 mV; ΔV_1/2_ = −2.2 ± 0.5 mV, *n* = 5; Figures 2B,D). No effects of 8-Br-cGMP and ODQ were found on I_h_ current density in both strains (data not shown).

Next, we manipulated intracellular cAMP levels by applying different concentrations of 8-Br-cAMP via the patch pipette. This resulted in a dose-dependent depolarizing shift in voltage-dependent activation (Figure 2E) accompanied by reduced activation time constants (Figure 2F) of I_h_ in both strains. ΔV_1/2_ revealed significant changes during application of 1 μM (ΔV_1/2_: WT = 8.5 ± 0.9 mV, *n* = 10; NO-GC2^−/−^ = 17.1 ± 1.6 mV, *n* = 8; *P* < 0.05) and 10 μM 8-Br-cAMP (ΔV_1/2_: WT = 17.4 ± 1.6 mV, *n* = 11; NO-GC2^−/−^ = 21.9 ± 1.7 mV, *n* = 8; *P* < 0.05; Figure 2D). Preincubation of slices with 200 μM SQ 22536 shifted activation potentials of I_h_ to more negative values in both strains. The effect was stronger in WT (V_1/2_ = −95.5 ± 0.6 ΔV_1/2_ = −9.1 ± 0.6 mV, *n* = 11), compared to NO-GC2^−/−^ mice (V_1/2_ = −95.2 ± 0.8 ΔV_1/2_ = −3.5 ± 0.7 mV, *n* = 10; *P*’s < 0.05; Figures 2D,E). 8-Br-cAMP and SQ 22536 did not change I_h_ current density in TC neurons (data is not shown).

More recently, the modulation of HCN2 and HCN4 channels by cCMP and cUMP has been reported (Zong et al., 2012). Both channel isoforms are major constituents of I_h_ in murine TC neurons (Ludwig et al., 2003; Leist et al., 2016). Thus, we examined the effect of cCMP and cUMP on I_h_ currents in WT neurons. cCMP is a partial agonist of HCN channels with an EC_50_ value of ~30 μM compared to 1 μM for cAMP (Zong et al., 2012). Therefore, we applied 30 μM cCMP and cUMP. Both compounds induced depolarizing shifts of V_1/2_ in TC compared to control cells (cCMP: V_1/2_ = −77.44 ± 2.3 mV; ΔV_1/2_ = 8.9 ± 2.3 mV; *n* = 6; *P’*s < 0.05); cUMP: V_1/2_ = −78.9 ± 3.6 mV, ΔV_1/2_ = 7.6 ± 3.5 mV; *n* = 6; *P’*s < 0.05; Figures 2D,G).

In order to allow better comparison between voltage and current clamp recordings and to assess potential effects of pH buffering conditions, we compared I_h_ current from WT TC neurons in NaHCO_3_ and HEPES buffered extracellular solutions in the presence of 0.5 mM BaCl_2_. Half maximal activation potential of I_h_ (NaHCO_3_: V_1/2_ = −89.7 ± 0.8 mV, *n* = 6; HEPES: V_1/2_ = −88.2 ± 1.2 mV, *n* = 5; *P* > 0.05) and current density (NaHCO_3_: I_h_ = 5.2 ± 0.6 pA/pF; HEPES: I_h_ = 6.9 ± 1.2 pA/pF, *n* = 5; *P* > 0.05) did not differ between the two recording conditions (data not shown).

**Figure 3:**
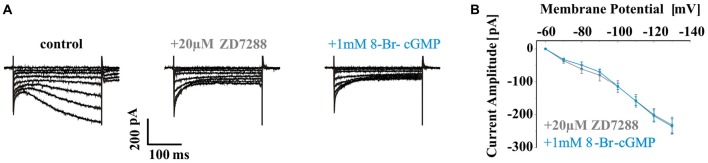
Effects of 8-Br-cGMP on WT inwardly rectifying potassium (I_KIR_) current. **(A)** Representative current traces evoked by increasing hyperpolarizing voltage steps (from −60 mV to −130 mV with 10 mV increments) of WT TC neurons under control condition (left panel; no blocker present) and in the presence of 20 μM ZD7288 (middle panel) and additional application of 1 mM 8-Br-cGMP (right panel). Typical I_KIR_ was seen in 20 μM ZD7288. **(B)** I_KIR_ amplitudes elicited by each voltage steps were plotted as I–V curves for different recording conditions as indicated. Please note that 8-Br-cGMP did not change the amplitude of I_KIR_ in dorsal lateral geniculate nucleus (dLGN) TC neurons.

Since Kir channels have been found to be modulated by cyclic nucleotides and are important in setting the RMP and determining the firing pattern of neurons, the effect of 8-Br-cGMP was studied on WT inwardly rectifying potassium (I_KIR_) current. I_KIR_ was evoked by hyperpolarizing voltage steps and was isolated from I_h_ by applying 20 μM ZD7288. Extracellular application of 1 mM 8-Br-cGMP did not change the amplitude of I_KIR_ (ZD7288: 231.9 ± 23.9 pA at −130 mV, *n* = 6; 8-Br-cGMP: 235.8 ± 22.8 pA, *n* = 6; *P* > 0.05; Figures 3A,B) in dLGN TC neurons.

### Influence of cGMP on TC Neurons Firing Patterns

**Figure 4:**
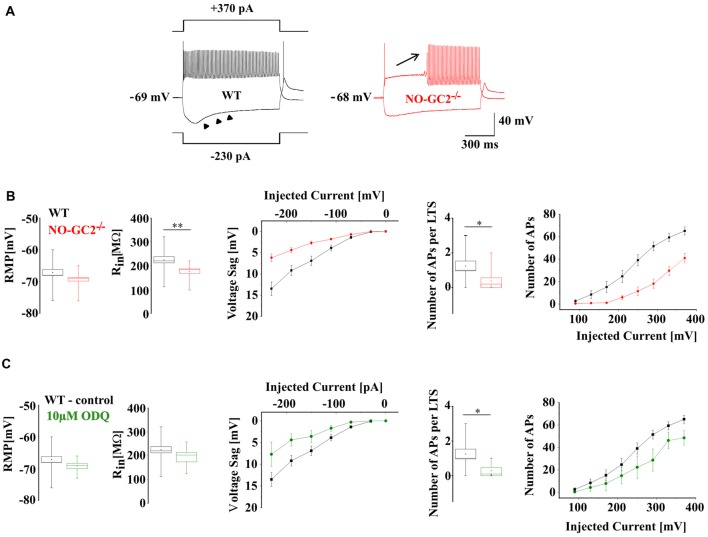
Passive and active membrane properties of TC neurons in WT and NO-GC2^−/−^. **(A)** Voltage responses of TC neurons recorded under current clamp conditions at rest. Both WT (black) and NO-GC2^−/−^ (red) neurons were injected with a series of hyperpolarizing and depolarizing currents. **(B)** Box and whisker plot showing the resting membrane potential (RMP) and R_in_ of NO-GC2^−/−^ (red box) and WT (black box) TC neurons. The amplitude of anomalous rectification induced by hyperpolarizing current injection was less pronounced in NO-GC2^−/−^ (red symbols) compared to WT (black symbols) mice. Upon release from membrane hyperpolarization the occurrence of low-threshold Ca^2+^ spikes (LTS) and the number of action potentials per LTS was higher in WT neurons (black box) in comparison to NO-GC2^−/−^ (red box). Depolarizing currents evoked less number of action potentials in NO-GC2^−/−^ (red symbols) compared to WT (black symbols) neurons. **(C)** Preincubation of WT slices in 10 μM ODQ (green box) did not change either RMP nor R_in_, compared to control conditions (black box). Voltage sag amplitudes were nominally reduced, occurrence of LTS and the number of action potentials per LTS was less pronounced and the number of APs in response to depolarizing current injections was nominally decreased after the blockage of GC (green symbols) in comparison to control (black symbols). *** indicate *P* < 0.05, *P* < 0.01, respectively.

To determine the functional impact of NO-GC2 activity on passive membrane properties and neuronal firing, TC neurons were recorded under current clamp conditions from the RMP (Figure 4A). While the RMP (WT: −67.1 ± 0.9 mV, *n* = 16; NO-GC2^−/−^: −69.3 ± 0.9 mV, *n* = 14; *P* > 0.05) did not differ between the strains, R_in_ was significantly reduced in NO-GC2^−/−^ (WT: 224.9 ± 13.1 MΩ, *n* = 16; NO-GC2^−/−^: 175.9 ± 8.7 MΩ, *n* = 14; *P* < 0.05; Figure 4B). Negative current injections (−230 to −30 pA, 1 s duration) induced significantly smaller voltage sags in the absence of NO-GC2 (WT: 13.5 ± 1.6 mV at −230 pA, *n* = 16; NO-GC2^−/−^: 6.2 ± 0.9 mV at −230 pA, *n* = 14; *P* < 0.05; Figures 4A,B). Upon release from the hyperpolarizing pulse NO-GC2^−/−^ mice generated less rebound low-threshold Ca^2+^ spikes (LTS) with action potentials ridding on top of it. Out of 16 WT cells 12 cells showed an LTS which triggered action potentials (number of action potentials per LTS: 1.25 ± 0.3, *n* = 16), whereas only 4 out of 14 NO-GC2-deficient cells displayed an LTS triggering action potentials (number of action potentials per LTS: 0.38 ± 0.2, *n* = 14; *P* < 0.05; Figure 4B). When TC neurons from NO-GC2^−/−^ mice were challenged with depolarizing currents (10–370 pA, 1 s duration), tonic activity was characterized by less APs compared to WT mice (WT: 65.1 ± 3.6 at 370 pA, *n* = 16; NO-GC2^−/−^: 40.9 ± 4.3 at 370 pA, *n* = 14; *P* < 0.05; Figures 4A,B). Preincubation of slices from WT in 10 μM ODQ was performed to mimic the effects of NO-GC2 deficiency under current clamp conditions. No changes in the RMP (control: −67.1 ± 0.9 mV, *n* = 16; 10 μM ODQ: −69.1 ± 0.9 mV, *n* = 7; *P* > 0.05) and R_in_ (control: 224.9 ± 13.1 MΩ, *n* = 16; 10 μM ODQ: 193.6 ± 19.6 MΩ, *n* = 7; *P* > 0.05) were found (Figure 4C). Voltage sag amplitudes were marginally decreased (control: 13.5 ± 1.6 mV at −230 pA, *n* = 16; 10 μM ODQ: 7.7 ± 2.8 mV, *n* = 7; *P* > 0.05), generation of LTS was less pronounced (number of action potentials per LTS: control: 1.25 ± 0.3, *n* = 16; 10 μM ODQ: 0.29 ± 0.2, *n* = 7; *P* < 0.05; Figure 4C) and number of APs was reduced in TC cells treated with ODQ (control: 65.1 ± 3.6 at 370 pA, *n* = 16; ODQ: 48.5 ± 6.4 at 370 pA, *n* = 7; *P* < 0.05; Figure 4C).

**Figure 5:**
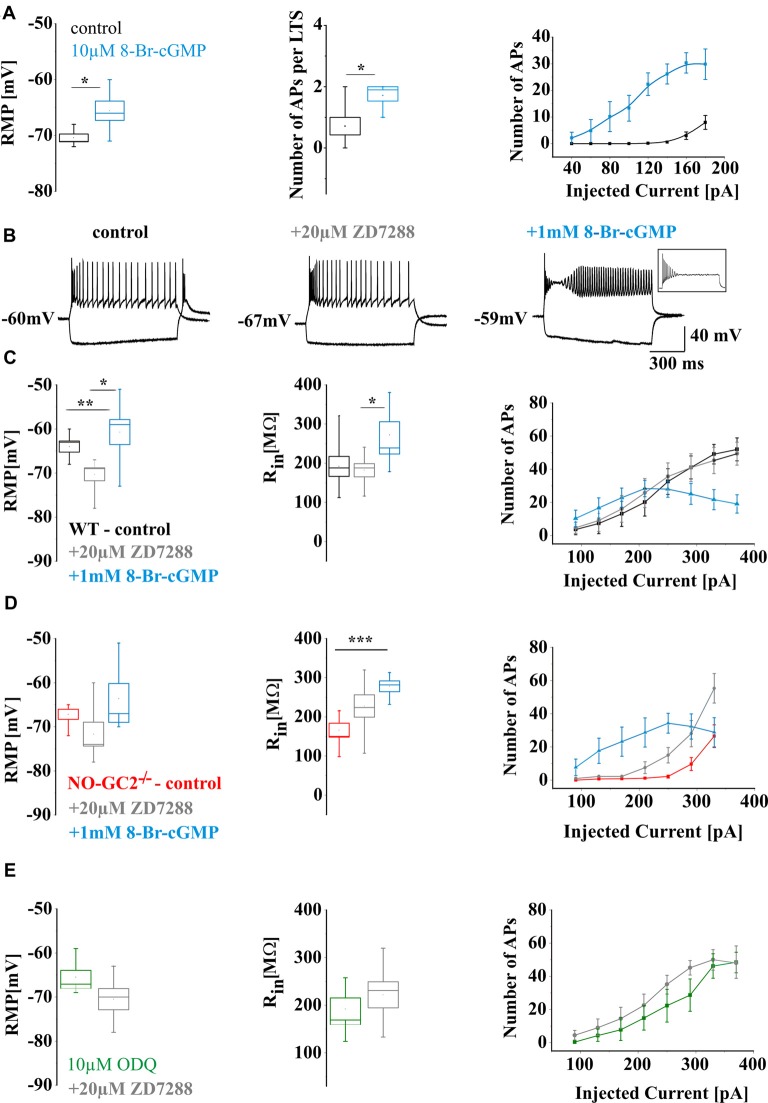
Effects of increased intracellular levels of cGMP on the electrical properties of WT and NO-GC2^−/−^ mice TC neurons. **(A)** Perfusion of WT TC neurons with an intracellular solution containing 10 μM 8-Br-cGMP. Left panel: the RMP of control TC cells (black box) and the cells treated by 10 μM 8-Br-cGMP (blue box). Middle panel: the number of APs per LTS induced upon release from the hyperpolarizing pulse in control (black box) and 10 μM 8-Br-cGMP (blue box) infused cells. Right panel: the number of APs induced by positive current injection in control (black symbols) and 10 μM 8-Br-cGMP (blue symbols) treated cells. **(B)** Example traces of WT neurons at −230 pA and 210 pA current injection recorded under control (black heading) condition and in the presence of 20 μM ZD-7288 (gray heading) and 1 mM 8-Br-cGMP (blue heading). Inset: please notice an example of depolarization block with 250 pA depolarizing current injection. **(C)** The passive and active membrane properties of WT neurons: the RMP (left panel), R_in_ (middle panel) and the number of APs (right panel) under control conditions (black box, symbols) and in the presence of 20 μM ZD-7288 (gray box, symbols) and 1 mM 8-Br-cGMP (blue box, symbols). **(D)** The passive and active membrane properties of NO-GC2 deficient neurons: the RMP (left panel), R_in_ (middle panel) and the number of APs (right panel) under control conditions (red box, symbols) and in the presence of 20 μM ZD-7288 (gray box, symbols) and 1 mM 8-Br-cGMP (blue box, symbols). **(E)** The RMP (left panel), R_in_ (middle panel) and the number of APs (right panel) of WT neurons incubated in 10 μM ODQ (green box, symbols) and in the presence of 20 μM ZD7288 (gray box, symbols). *,**,*** indicate *P* < 0.05, *P* < 0.01, *P* < 0.001, respectively.

Next, effects of exogenously increased cGMP concentrations were assessed. Application of 10 μM 8-Br-cGMP via the patch pipette in WT TC neurons resulted in a more positive RMP (control: −70.43 ± 0.69 mV, *n* = 7; cGMP: −65.57 ± 1.7 mV, *n* = 7; *P* < 0.05; Figure 5A) and increased occurrence of LTS. All cGMP treated cells showed LTS (number of action potentials per LTS: control: 0.7 ± 0.3, *n* = 7; 10 μM 8-Br-cGMP: 1.7 ± 1.8, *n* = 7;* P* < 0.05; Figure 5A). Under these conditions, TC neurons fired significantly more tonic APs (Figure 5A). To assess to what extend these changes may be related to modulation of I_h_, neurons from WT and NO-GC2^−/−^ mice were washed with the HCN channel blocker ZD7288 (20 μM) and then subjected to an extracellular application of 8-Br-cGMP (1 mM; Figure 5B). In all cells tested, ZD7288 completely abolished the voltage sag. In WT, ZD7288 strongly hyperpolarized the RMP (control: −64 ± 1.3 mV, *n* = 7; ZD7288: −70.3 ± 1.5 mV, *n* = 7; *P* < 0.05), while 8-Br-cGMP caused depolarization of the RMP (8-Br-cGMP: −60.7 ± 2.8 mV, *n* = 7; *P* < 0.05, Figure 5C). Although ZD7288 did not change the R_in_ of WT cells (control: 191.9 ± 25.4 MΩ, *n* = 7; ZD7288: 182.2 ± 16.9 MΩ, *n* = 7; *P* > 0.05), administration of 8-Br-cGMP strongly increased R_in_ to 272.3 ± 33.4 MΩ (*n* = 7; *P* < 0.05; Figure 5C). Tonic firing was only nominally increased after the application of ZD7288 (Figure 5C). With injected currents up to 90 pA, WT TC neurons fired slightly more APs after additional application of 8-Br-cGMP. Stronger current injections however were associated with partial depolarization block (Figure 5B; inset), thereby pointing to the loss of hyperpolarizing influences (Figures 5B,C). When hyperpolarizing K^+^ currents are reduced, neurons may enter the state of depolarization block where under sustained input current of increasing strength neurons eventually reduce or stop firing, while membrane potential fluctuations above threshold are present (Bianchi et al., 2012). In NO-GC2^−/−^ the direction of changes in RMP induced by ZD7288 and 8-Br-cGMP was similar to WT (control: −67.2 ± 1.1 mV, *n* = 6; ZD7288: −71.7 ± 2.7 mV, *n* = 6; 8-Br-cGMP: −63.6 ± 3.7 mV, *n* = 6; *P’*s > 0.05; Figure 5D). Both compounds increased the R_in_ of NO-GC2-deficient cells (control: 165.9 ± 17.7 MΩ, *n* = 6; ZD7288: 227.7 ± 28.4 MΩ, *n* = 6, *P* > 0.05; 8-Br-cGMP: 278 ± 16.9 MΩ, *n* = 6; *P* < 0.05; Figure 5D). Both ZD7288 and 8-Br-cGMP increased the excitability of NO-GC2^−/−^ TC neurons, compared to control recordings. Again, AP firing in the presence of both compounds was characterized by a partial depolarization block (Figure 5D). RMP, R_in_ and firing did not differ between the strains after ZD7288 and cGMP application (Figures 5C,D). When WT slices were preincubated with 10 μM ODQ, application of ZD7288 (20 μM) marginally hyperpolarized the RMP (ODQ: −65.5 ± 1.6 mV, *n* = 6; ZD7288: −70.5 ± 2.4 mV, *n* = 6; *P* > 0.05) and increased the R_in_ (ODQ: 192.1 ± 23.2 MΩ, *n* = 6; ZD7288: 221.9 ± 27.3 MΩ, *n* = 6;* P* > 0.05) of TC. The number of spikes with depolarizing current injections was slightly increased after the application of ZD7288 (Figure 5E).

### Effect of Ba^2+^ on Membrane Properties of TC Neurons

**Figure 6:**
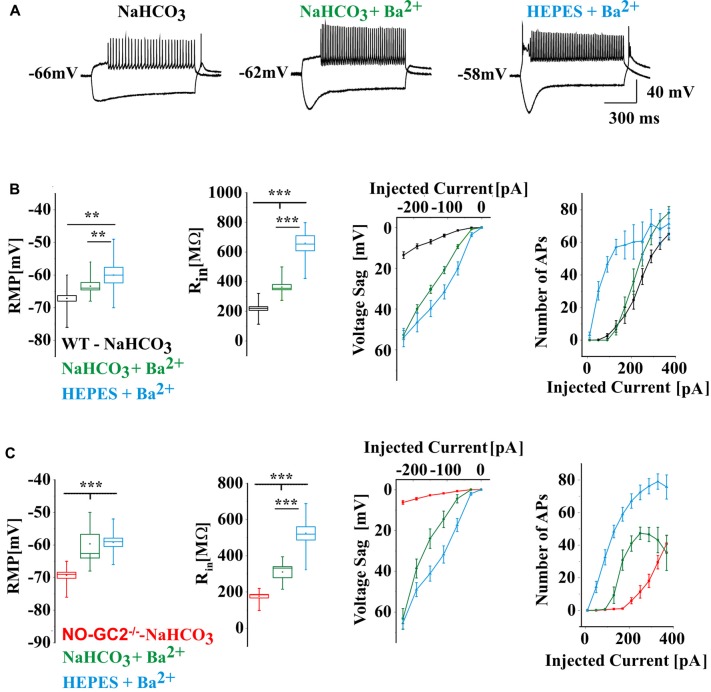
**(A)** Example traces of WT current clamp recordings at −230 pA and 210 pA current injections. The recordings were done in bicarbonate (NaHCO_3_) buffered, Ba^2+^ free extracellular solution (black heading), in bicarbonate buffered solution contained 0.5 mM BaCl_2_ (green heading) and in HEPES buffered solution contained 0.5 mM BaCl_2_ (blue heading). **(B)** Active and passive membrane properties of WT cells recorded in different extracellular solutions. **(C)** Active and passive membrane properties of NO-GC2^−/−^ cells recorded in different extracellular solutions. **,*** indicate *P* < 0.01, *P* < 0.001, respectively.

In order to allow better comparison between current and voltage clamp recordings and to assess the contribution of Ba^2+^-sensitive inward rectifier and K_2P_ channels, like TASK and TREK channels, BaCl_2_ (0.5 mM) was added to NaHCO_3_- and HEPES-buffered extracellular solutions. Results were compared to the data described above (i.e., Ba^2+^ free NaHCO_3_-buffered solution; Figure 6). In both genotypes Ba^2+^ significantly changed passive and active membrane properties of TC neurons. In the presence of Ba^2+^, the RMP was strongly depolarized, R_in_ was increased, voltage sags were unmasked, and the number of APs was significantly increased (Figures 6A–C). In the presence of Ba^2+^ voltage sag amplitudes were significantly larger in NO-GC2^−/−^ in comparison to WT (NaHCO_3_ + Ba^2+^: WT: 52.74 ± 2.25 mV at −230 pA, *n* = 12; NO-GC2^−/−^: 63.38 ± 5 mV at −230 pA, *n* = 6; HEPES: WT: 53.95 ± 4.48 mV at −230 pA, *n* = 7; NO-GC2^−/−^: 65.45 ± 2.8 mV at −230 pA, *n* = 10, *P’*s < 0.05; Figures 6B,C).

**Figure 7:**
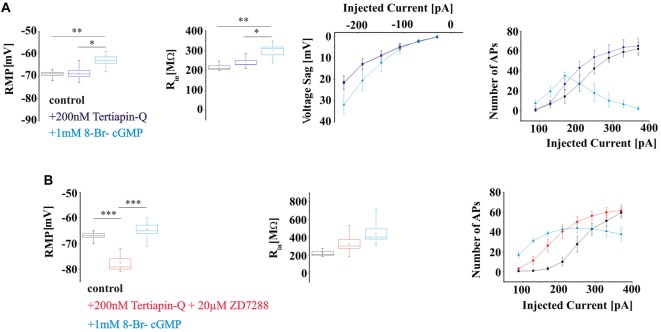
Effects of elevated 8-Br-cGMP levels on electrical properties of WT TC neurons in the presence of Kir and HCN channels blockers. **(A)** Passive and active properties of TC in control (black) and after the application of Kir channels blocker Tertiapin-Q (lilac) and 8-Br-cGMP (blue). **(B)** Effects of 8-Br-cGMP on electrical properties of TC in the presence of Kir and HCN channels blockers (red). *,**,*** indicate *P* < 0.05, *P* < 0.01, *P* < 0.001, respectively.

To assess the possible contribution of I_KIR_ to hyperpolarization-induced voltage deflections and firing pattern of TC neurons, WT slices were washed for 15 min with the Kir channel inhibitor Tertiapin-Q, thereafter effects of extracellular application of 8-Br-cGMP on membrane properties were studied. Tertiapin-Q (200 nM) did not change either the RMP (control: −69 ± 0.7 mV, *n* = 6; Tertiapin-Q: −68.8 ± 1.4 mV, *n* = 6; *P* > 0.05), nor R_in_ (control: 215.5 ± 7.3 MΩ, *n* = 6; Tertiapin-Q: 239.4 ± 9.9 MΩ; *P* > 0.05) and voltage sag amplitudes (control: 21.3 ± 2.9 mV at −230 pA, *n* = 6; Tertiapin-Q: 21.8 ± 3.6 mV at −230 pA, *n* = 6; *P* > 0.05; Figure 7A) of TC neurons. Application of 8-Br-cGMP significantly depolarized the RMP (8-Br-cGMP: −62.8 ± 1.4 mV;* P* < 0.05), increased R_in_ (8-Br-cGMP: 297.4 ± 19.4 MΩ, *n* = 6; *P* < 0.05) and voltage sag amplitudes (8-Br-cGMP: 31.9 ± 4.7 mV, *n* = 6; *P* < 0.05; Figure 7A). Firing was slightly increased by Tertiapin-Q and 8-Br-cGMP caused further excitation of TC neurons, eventually resulting in a depolarization block with higher depolarizing current injections (Figure 7A).

Next, the effects of elevated levels of 8-Br-cGMP on TC neurons membrane properties were studied in the presence of Kir and HCN channels inhibitors Tertiapin-Q and ZD7288, respectively (Figure 7B). Co-administration of the two inhibitors significantly hyperpolarized the RMP (control: −66.8 ± 0.7 mV, *n* = 6; Tertiapin-Q + ZD7288: −77.5 ± 1.6 mV, *n* = 6; *P* < 0.05) and nominally increased R_in_ (control: 228.3 ± 0.7 MΩ: Tertiapin-Q + ZD7288: 325.6 ± 48.8 MΩ, *n* = 6; *P* > 0.05; Figure 7B) of TC neurons. The RMP was significantly depolarized (8-Br-cGMP: −64.5 ± 1.7 mV, *n* = 6; *P* < 0.05) and R_in_ was increased (8-Br-cGMP: 438.4 ± 57.7 MΩ, *n* = 6; *P* > 0.05) following extracellular application of 1 mM 8-Br-cGMP. Inhibition of Kir and HCN channels resulted in an increased number of APs with small depolarizing current injections and the occurrence of a depolarization block was noticed in the presence of 8-Br-cGMP in TC neurons (Figure 7B).

### GC Activity in dLGN

**Figure 8:**
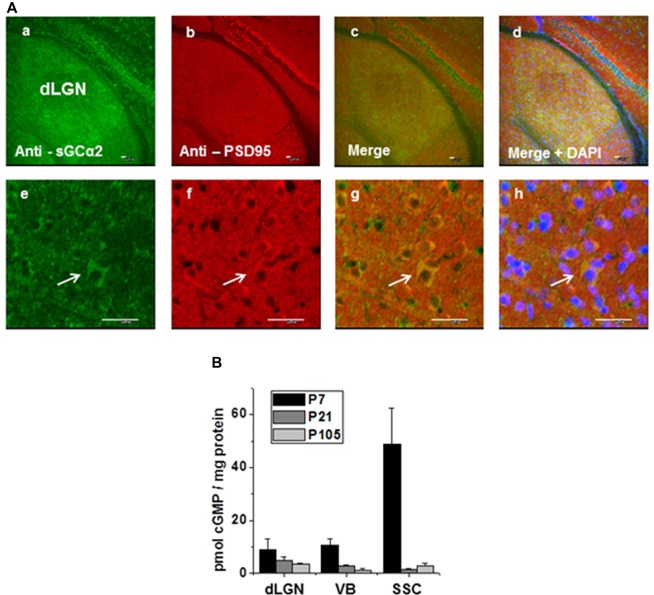
Immunohistochemical staining of NO-GCα2, postsynaptic density protein 95 (PSD95) in dLGN of WT mice. **(A)** Immunoreactivity of triple-stained thalamic tissue with antibodies against the α2 subunit of NO-GC2 (green), postsynaptic density protein, PSD 95 (red) and nuclear marker, DAPI (blue) is shown in lower **(a,b,c,d)** and higher **(e,f,g,h)** magnification. Scale bars = 25 μm. **(B)** Quantification of basal cGMP levels (pmol cGMP/mg protein) at different postnatal ages in the dLGN, ventrobasal thalamus (VB) and somatosensory cortex (SSC).

The presence and localization of NO-GC2 in the thalamus was analyzed immunohistochemically. A strong fluorescent signal was detected in dLGN (Figure 8A). Higher magnification images revealed expression in the cellular boundaries. Co-staining for NO-GCα2 and the PSD95 revealed a strong overlap of the fluorescent signals, pointing to a postsynaptic localization.

In order to address a potential contribution of cGMP in TC neurons signaling, we quantified the amount of cGMP in tissue from different brain areas at different developmental stages. In all samples (dLGN, VB, SSC) the highest cGMP levels were consistently detected in young animals (postnatal day 7, P7) with the SSC containing the largest amount of cGMP. During postnatal development (P21 and P107) the tissue contained lower cGMP levels (Figure 8B).

### Reduction of Burst Activity in the Thalamic Network in the Absence of NO-GC2

**Figure 9:**
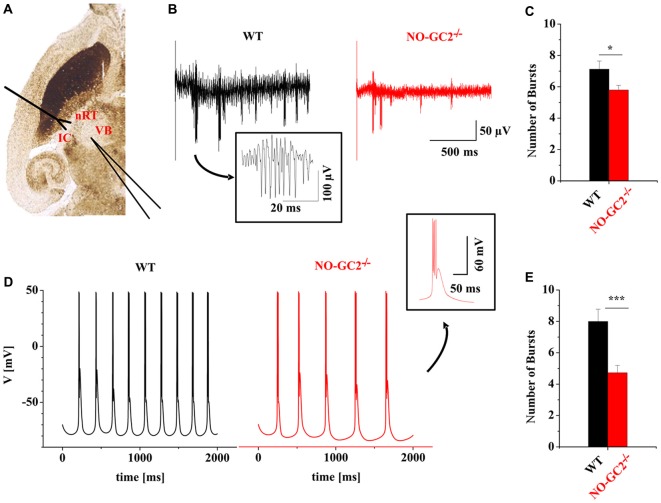
Dysfunction of the NO-GC2 signaling pathway changes thalamic oscillations. **(A)** Dampened rhythmic activity was induced by exciting neurons in the reticular thalamus (nRT) via stimulation of the internal capsule (IC) in horizontal thalamic slices. Neuronal responses were measured in the ventrobasal thalamus (VB). **(B)** Example traces of rhythmic burst activities from WT (black) and NO-GC2^−/−^ (red) mice. **(C)** The number of bursts (red bar) was significantly decreased in NO-GC2^−/−^ compared to WT (black bar) mice. **(D)** Computer simulation of rhythmic activity in a mathematical model. In this simulation two nRT neurons reciprocally communicate via GABA_A_-mediated connections and project to two TC neurons via GABA_A_ and GABA_B_ signaling. The feedback from TC neurons is carried by AMPA receptors to both nRT neurons. Rhythmic bursting profiles with I_h_ properties taken from TC neurons of WT (black) and NO-GC2^−/−^ (red) mice are shown. **(E)** With I_h_ parameters set to the values obtained from TC cells of WT (black bar) and NO-GC2^−/−^ (red bar) mice, the number of bursts was significantly lower in NO-GC2^−/−^ mice. *,*** indicate *P* < 0.05, *P* < 0.001, respectively.

The intrathalamic network activity is involved in the generation of different thalamic oscillations such as sleep spindle and delta oscillations which critically depend on activation of I_h_ (Kanyshkova et al., 2009, 2012). Rhythmic bursting is a property of thalamic cells and has frequently been used as a measure of intrathalamic rhythmicity in horizontal thalamic slices conserving axonal connections between nRT and VB TC neurons (Huguenard and Prince, 1994; Yue and Huguenard, 2001). In this model system intrathalamic oscillations are generated by reciprocal interactions between inhibitory nRT neurons and excitatory TC neurons. Stimulation of nRT neurons evokes IPSPs in VB neurons and triggers rebound burst activity in TC. Rebound bursts of TC neurons re-excite the nRT and the cycle starts again. Here dampened oscillatory activity (i.e., up to 6–8 cycles) was induced by stimulation of the IC (containing TC and corticothalamic fibers) and recorded in VB (Figure 9A). Compared to control animals, NO-GC2^−/−^ mice revealed a significantly lower number of bursts in response to a single stimulus (NO-GC2^−/−^: 5.8 ± 0.3 bursts, *n* = 12; WT: 7.1 ± 0.5 bursts, *n* = 8; *P* < 0.05; Figures 9B,C).

To further assess the influence of NO-GC2 activity on rhythmic activity in the thalamic network, a mathematical modeling approach was used (Kanyshkova et al., 2009, 2012; Figure 9D). Spontaneous rhythmic bursting was analyzed in an interconnected four-cell model of two TC and two nRT neurons which was used for simulation of the intrathalamic network activity (Kanyshkova et al., 2009, 2012). Compared to WT, the number of bursts generated within the stimulation period of 2 s based on NO-GC2^−/−^-derived parameters was significantly lower (NO-GC2^−/−^: 4.7 ± 0.5 bursts, *n* = 11; WT: 8.0 ± 0.8 bursts, *n* = 11; *P* < 0.05; Figure 9E).

### Spectral Power Characteristics of NO-GC2^−/−^ Mice During Different Behavioral States

**Figure 10:**
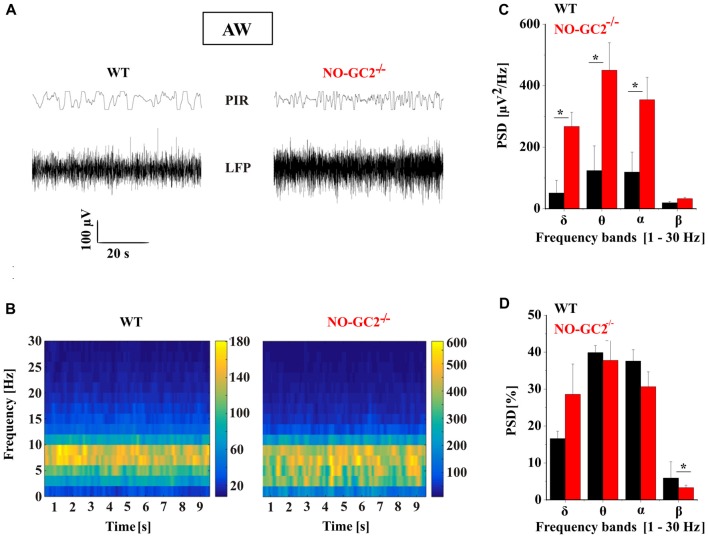
Effect of NO-GC2-deficiency on active waking (AW) state. **(A)** Raw local field potential (LFP) example traces during AW from SSC and movement detector (Passive Infrared Recording System, PIR) in WT (left) and NO-GC2^−/−^(right) mice. **(B)** The mean power spectrum of the LFPs between 1 Hz and 30 Hz obtained from 20 epochs of 10 s duration analyzed during the first 2 h of the light on period. **(C)** The mean values of raw PSD during AW showed enhanced δ (1–4 Hz), θ (5–8 Hz) and α (9–12 Hz) activity in NO-GC2^−/−^ (red bar) compared to WT (black bar) mice. **(D)** The normalized PSD values are presented as percentage for WT (black bar) and NO-GC2^−/−^ (red bar) mice. *P < 0.05.

**Figure 11:**
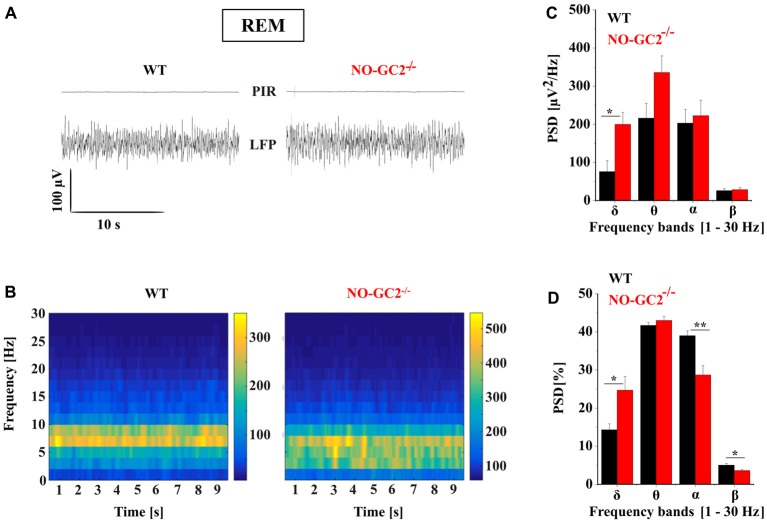
Effect of NO-GC2-deficiency on rapid eye movement (REM) sleep. **(A)** Raw LFP example traces during REM sleep in WT (left) and NO-GC2^−/−^ (right) mice. Note, during REM sleep no movements are detected on PIR, indicating the immobility of the animal. **(B)** LFP power spectrum of different frequencies (1–30 Hz) over time. **(C)** During REM sleep, electroencephalogram (EEG) spectral power density is increased in the δ (1–4 Hz) and θ (5–8 Hz) band in NO-GC2^−/−^(red bar) compared to WT (black bar) mice. **(D)** The normalized PSD values are presented as percentage in WT (black bar) and NO-GC2^−/−^ (red bar) mice. *,** indicate *P* < 0.05, *P* < 0.01, respectively.

Thalamic bursting is involved in the generation of TC rhythms such as delta oscillations which are found on the LFP and EEG recordings during SWS sleep and anesthesia (Steriade et al., 1993; Timofeev, 2011). Therefore, we assessed the effects of NO-GC2 deficiency on cortical oscillations by performing LFP recordings from the SSC of freely moving mice (Figures 10, 11). Recordings made during the first 2 h of the light period were taken for analysis. Absolute spectral power of LFP signals recorded from NO-GC2^−/−^ (*n* = 4) and WT (*n* = 5) mice was assessed for the delta, theta, alpha and beta bands and compared between strains during the four different behavioral states (AW, REM, LSWS and DSWS).

During AW both strains show predominant theta activity in the LFP, characteristic for a moving rodent (Figure 10A). NO-GC2^−/−^ mice displayed more power than WT mice within the δ (WT: 51 ± 9.5 μV^2^/Hz; NO-GC2^−/−^: 267 ± 41 μV^2^/Hz; *P* < 0.05), θ (WT: = 132 ± 80 μV^2^/Hz; NO-GC2^−/−^: 450 ± 89 μV^2^/Hz; *P* < 0.05) as well as within the α frequency band (WT: 119 ± 64 μV^2^/Hz; NO-GC2^−/−^: 354 ± 72 μV^2^/Hz; *P* < 0.05), whereas the β power did not significantly differ between the strains (WT: = 19 ± 4 μV^2^/Hz; NO-GC2^−/−^: 32 ± 4 μV^2^/Hz; *P* > 0.05; Figures 10B,C). The percentage of EEG frequencies (%) were nominally enhanced in δ (WT: 16.6 ± 2.2%; NO-GC2^−/−^: 28.6 ± 8.2%; *P* > 0.05) and significantly decreased in β frequency range in NO-GC2^−/−^ (3.3 ± 0.69%), compared to WT (5.9 ± 0.44%; *P* < 0.05, Figure 10D) mice.

Both strains show a typical REM sleep spectrum, characterized by the presence of theta activity in the LFP (Figure 11A). NO-GC2^−/−^ mice had a significantly higher spectral power than WT mice in the δ band during REM sleep (WT: 76 ± 28 μV^2^/Hz; NO-GC2^−/−^: 199 ± 31 μV^2^/Hz; *P* < 0.05). However, strains did not differ in power of θ, α and β frequency bands (Figures 11B,C). The normalized values of different frequencies showed increased δ (WT: 14.3 ± 1.58%; NO-GC2^−/−^: 24.7 ± 3.62%; *P* < 0.05) and decreased α (WT: 38.92 ± 1.2%; NO-GC2^−/−^: 28.64 ± 2.4%; *P* < 0.05) and β (WT: 5 ± 0.42%; *P* < 0.05; NO-GC2^−/−^: 3.58 ± 0.33%; Figure 11D) activities in NO-GC2^−/−^ mice.

During LSWS and DSWS there were no strain differences in any of the bands of the raw LFPs, as well as in the normalized data (data not shown).

## Discussion

The modulation of I_h_ in TC neurons of different mammalian species by cAMP is well established (McCormick and Pape, 1990; Budde et al., 2005; Leist et al., 2016). Fewer studies have addressed effects mediated by cGMP. While the increase in current and the shift to depolarized potentials in the activation curve by NO donors and cGMP as well as the involvement of GC activity in I_h_ modulation have been described before (Pape and Mager, 1992; Yang and Cox, 2008), the GC subtype involved in these phenomena has not been yet identified. Experimental data suggested low to moderate mRNA expression of GC α_1_, α_2_ and β_1_ subunits which form the heterodimeric NO-GC1 (α_1_β_1_) and NO-GC2 (α_2_β_1_) isoforms in different thalamic nuclei (Giuili et al., 1994; Gibb and Garthwaite, 2001). Notably, V_1/2_ of I_h_ in TC neurons of NO-GC2^−/−^ mice was found to be more hyperpolarized compared to WT, indicating that the current is controlled by the basal activity of postsynaptic NO-GC2. The control of I_h_ by basal cGMP production is in good agreement with electrophysiological analyses of CA1 pyramidal neurons (Neitz et al., 2014).

It has recently been suggested that cCMP and cUMP should also be considered in HCN channel-regulated processes (Zong et al., 2012) and soluble GC might also synthesize cCMP and cUMP in the presence of NO and Mn^2+^ (Beste et al., 2012). In agreement with this previous notion, we found that I_h_ in native neurons is moderately modulated by cCMP and cUMP.

In other neuronal cell types an increase in AP firing induced by NO donors which was sensitive to ODQ has been previously observed, suggesting participation of cGMP-dependent mechanisms (Kim et al., 2004). Intracellular application of 8-Br-cGMP strongly increased tonic firing in WT TC neurons and a number of distinct changes in electrophysiological properties were found in TC neurons following knock out or inhibition of NO-GC2. While NO-GC2^−/−^ mice revealed reduced voltage sag amplitudes and tonic firing, RMP was unchanged. With respect to the number of triggered action potentials, LTS firing was less pronounced in NO-GC2-deficient mice. It has been shown before that the voltage-dependent properties of I_h_ crucially influence LTS generation and burst firing in TC neurons (Hughes et al., 1998). Positive shifts in the voltage-dependency of I_h_ increase the amplitude and duration of the LTS while negative shifts have opposite effects. Thus in line with our findings the reduced availability of I_h_ is associated with a decreased number of action potentials in the LTS.

Although we registered a decreased availability of I_h_ in TC neurons of NO-GC2^−/−^ mice which is generally in line with some of our functional findings, the intrinsic membrane properties of TC neurons are expected to depend on several membrane currents. Setting of the RMP (Meuth et al., 2006), rhythmic bursting (Amarillo et al., 2014) and tonic firing (Kasten et al., 2007) of rodent TC neurons is based on the dynamic interaction of multiple membrane currents, with some of them being modulated by cGMP in a differential manner. While HCN channels are activated by direct binding of cGMP to the cyclic nucleotide binding domain (CNBD), the situation is more complex when it comes to K_2P_ channels. PKG-dependent activation of TASK-1 and TREK-1 (Toyoda et al., 2010) as well as inhibition of heteromeric TASK-1/TASK-3 (Gonzalez-Forero et al., 2007) channels have been described. In addition, inward rectifier K^+^ currents have been shown to be inhibited in a cGMP-dependent manner (Dixon and Copenhagen, 1997) and a number of Kir channel subtypes (including members of the Kir2, Kir3 and Kir6 families) are expressed in thalamic cells (Thomzig et al., 2005). In TC neurons of different species several K_2P_ (TASK-1, TASK-3, TREK-1, TREK-2) and Kir channels (Kir2, Kir3) are expressed and have important contributions to the RMP, anomalous rectification and firing pattern (Meuth et al., 2003, 2006; Bista et al., 2015), thereby pointing to a complex scenario when cGMP-dependent effector functions are considered. The knockout of NO-GC2 and application of ODQ which both decrease the basal cGMP levels may have inhibited depolarizing HCN channels and (positively and negatively) modulated diverse hyperpolarizing K^+^ channels in a way that the net effects on RMP and R_in_ in the present study were inconspicuous. When taking the effects of exogenous cGMP application also into account, the strong effect of 8-Br-cGMP on RMP, R_in_ and tonic firing in the presence of ZD7288 (i.e., with HCN channels blocked) clearly points to the involvement of further ion channels. Here the combination of membrane depolarization and increased R_in_ is in line with the inhibition of TASK1/TASK3 heteromers which are present in TC neurons (Meuth et al., 2006). Indeed, application of Ba^2+^ which blocks TASK, TREK and Kir channels was associated with strong membrane depolarization, increased R_in_ and strongly enhanced tonic firing. Specific inhibition of some Kir channel types by Tertiapin-Q however, did not change the RMP and R_in_ in WT TC neurons. Moreover, extracellular application of 8-Br-cGMP had no influence on I_KIR_ amplitudes in Voltage clamp recordings. Taken together, the pharmacological manipulations indicate that altered modulation of cGMP-sensitive K_2P_ channels (Ma et al., 2011) may contribute to the phenotype of NO-GC2-deficient mice. But it does not exclude other ion channels as well.

Sleep is a complex process and controlled by several mechanisms (Borbély and Tobler, 2011) such as homeostatic, allostatic and circadian components. All regulators must ultimately target the thalamus to affect the cellular mechanisms that induce stable and global sleep-related oscillatory activity and allow the state-dependent gating of sensory information (Coulon et al., 2012). NO-dependent signaling is critically involved in the regulation of sleep homeostasis (Kalinchuk et al., 2006) and variations of NO and cGMP levels in the brain have been observed during the sleep-wake cycle (Kostin et al., 2013). We observed a strong increase in delta activity of the EEG as a consequence of NO-GC2 deficiency during AW and REM sleep. The nerve terminals of the mesopontine tegmentum cholinergic neurons which are part of the ascending reticular activating system have the ability to synthesis NO (Vincent, 2000). The release of NO from these thalamic afferents during arousal or REM sleep is followed by depolarization of TC neurons. It is possible that lack of postsynaptically located NO-GC2 receptors and decreased excitability of TC neurons in NO-GC2 mice disrupt the transition to AW and REM sleep and increases periods of slow oscillations in the EEG. The decreased availability to HCN channels may additionally hamper their pacemaker function and slow down oscillatory activity. We suggest that in the thalamus the cellular mechanisms of cGMP action may involve activation of HCN channels in addition to cGMP-dependent protein kinases. In addition, complete loss of the HCN2 channel gene and decreased responsiveness of I_h_ were associated with the appearance of pathological slow high amplitude oscillations (5–7 Hz) in the EEG in form of spike-and-wave discharges (Ludwig et al., 2003; Kuisle et al., 2006). These pathological activities were not found in NO-GC2^−/−^. Furthermore loss of HCN1 expression in the forebrain is associated with increased theta oscillations during AW and REM sleep (Nolan et al., 2004), and deletion of HCN channels auxiliary subunit TRIP8b increases delta oscillations during AW (Zobeiri et al., 2018). Therefore the effects of the NO-GC2 gene knockout are in agreement with reduced activation of I_h_ in forebrain neurons which result in increased slow frequency bands in EEG recordings.

The involvement of the NO/GC/cGMP pathway in the regulation of sleep and wakefulness are not fully conclusive and in part even contradictory (Cavas and Navarro, 2006). While some studies indicate that NOS inhibition increases SWS and NO is required for arousal (Burlet and Cespuglio, 1997), opposite responses and differential effects on REM and non-REM sleep have been described (Hars, 1999). Nevertheless, the use of NOS inhibitors, NO donors and 8-Br-cGMP in cats *in vivo* supported the role of cGMP in controlling rhythmic neuronal activity in thalamic and cortical neurons, which may play a role in sleep/wake transition (Cudeiro et al., 2000). Divergent findings might be due to variances in timing and dosage of drug administration, acute pharmacological treatment vs. genetic background (transgenic animals), and NO-independent cGMP signaling (Hess et al., 2005). The fact that cGMP-dependent signaling has multiple effectors throughout the brain which are difficult to control experimentally may have additionally contributed to the ongoing discussion (Russwurm et al., 2013).

Neurons of the nRT reveal intrinsic pacemaking properties and participate in intrathalamic network operations which allow them to generate and synchronize spindle waves (7–15 Hz), a hallmark of early sleep stages (Fuentealba and Steriade, 2005). Since nRT and TC neurons are interconnected in a loop, and we reported decreased LTS characteristics and damped burst activities *in vitro* from NO-GC2^−/−^ slice preparation, changed intrinsic properties of TC neurons may affect spindle oscillations. In addition, it was suggested that deactivation of I_h_ is important to terminate spindle activity (Bal and McCormick, 1996). It is therefore expected that the number or the shape of spindle waves is changed in NO-GC2^−/−^ animals. Analysis of the cellular effects of NO-GC2-deficiency in nRT neurons and combined electrophysiological recordings in nRT and VB *in vivo* may be well suited to investigate this aspect in future studies.

Based on our results, we conclude that thalamic HCN channels are modulated by different cyclic nucleotides and that cGMP is a good candidate to regulate intrathalamic and cortical activities. However, the action of cGMP is broad, involving complex signaling pathways and is thus not limited to the modulation of HCN channels. Since reduced basal levels of cGMP decrease the excitability of TC cells, our data are in agreement with previous studies and supports the idea that the role of cGMP in thalamus is excitatory. The increased occurrence of delta and theta band activity during AW characterizes the loss of NO-GC2 as a TC dysrhythmia syndrome (Llinás et al., 1999) and supports the view that slow oscillations are an intrinsic property of the TC system (Timofeev, 2011).

## Author Contributions

MD, RC, EM, AL, MZ, BB, SB and AB performed experiments and analyzed/interpreted data. TB designed and supervised the project and reviewed all experiments. H-CP, DK and GvL provided important scientific input. MD and TB wrote the manuscript. All authors edited and agreed on the final version of the manuscript.

## Conflict of Interest Statement

The authors declare that the research was conducted in the absence of any commercial or financial relationships that could be construed as a potential conflict of interest.

## Abbreviations

AW: active wakefulness
DSWS: deep slow wave sleep
EEG: electroencephalogram
HCN: hyperpolarization-activated cyclic nucleotide-gated channels
IC: internal capsule
I_h_: hyperpolarization activated current
LFP: local field potential
LSWS: light slow wave sleep
LTS: low-threshold Ca^2+^ spike
NO: nitric oxide
nonREM: non-rapid eye movement sleep
nRT: reticular thalamus
PIR: passive infrared recording system
REM: rapid eye movement sleep
Rin: input resistance
RMP: resting membrane potential
SSC: somatosensory cortex
SWS: slow wave sleep
TC: thalamocortical
V_1/2_: half maximal activation
VB: ventrobasal thalamus.

## References

[B1] Amarillo Y. Zagha E. Mato G. Rudy B. Nadal M. S. (2014). The interplay of seven subthreshold conductances controls the resting membrane potential and the oscillatory behavior of thalamocortical neurons. J. Neurophysiol. 112, 393–410. 10.1152/jn.00647.2013 24760784 PMC4064413

[B2] Backer O. D. Elinck E. Sips P. Buys E. Brouckaert P. Lefebvre R. A. (2008). Role of the soluble guanylyl cyclase α_1_/α_2_ subunits in the relaxant effect of CO and CORM-2 in murine gastric fundus. Neunyn Schmiedebergs Arch. Parmacol. 378, 493–502. 10.1007/s00210-008-0315-6 18563392

[B3] Bal T. McCormick D. A. (1996). What stops synchronized thalamocortical oscillations? Neuron 17, 297–308. 10.1016/s0896-6273(00)80161-0 8780653

[B4] Beste K. Y. Burhenne H. Kaever V. Stasch J.-P. Seifert R. (2012). Nucleotidyl cyclase activity of soluble guanylyl cyclase α_1_β_1_. Biochemistry 51, 194–204. 10.1021/bi201259y 22122229

[B5] Bianchi D. Marasco A. Limongiello A. Marchetti C. Marie H. Tirozzi B.. (2012). On the mechanisms underlying the depolarization block in the spiking dynamics of CA1 pyramidal neurons. J. Comput. Neurosci. 33, 207–225. 10.1007/s10827-012-0383-y 22310969

[B6] Bista P. Cerina M. Ehling P. Leist M. Pape H.-C. Meuth S. G.. (2015). The role of two-pore-domain background K^+^ (K_2_p) channels in the thalamus. Pflugers Arch. 467, 895–905. 10.1007/s00424-014-1632-x 25346156

[B7] Borbély A. A. Tobler I. (2011). Manifestations and functional implications of sleep homeostasis. Handb. Clin. Neurol. 98, 205–213. 10.1016/b978-0-444-52006-7.00013-7 21056188

[B10] Budde T. Caputi L. Kanyshkova T. Staak R. Abrahamczik C. Munsch T.. (2005). Impaired regulation of thalamic pacemaker channels through an imbalance of subunit expression in absence epilepsy. J. Neurosci. 25, 9871–9882. 10.1523/jneurosci.2590-05.2005 16251434 PMC6725576

[B11] Burlet S. Cespuglio R. (1997). Voltammetric detection of nitric oxide (NO) in the rat brain: its variations throughout the sleep-wake cycle. Neurosci. Lett. 226, 131–135. 10.1016/s0304-3940(97)00247-4 9159507

[B12] Cavas M. Navarro J. F. (2006). Effects of selective neuronal nitric oxide synthase inhibition on sleep and wakefulness in the rat. Prog. Neuro-Psychopharmacology Biol. Psychiatry 30, 56–67. 10.1016/j.pnpbp.2005.06.013 16023276

[B13] Coulon P. Budde T. Pape H.-C. (2012). The sleep relay–the role of the thalamus in central and decentral sleep regulation. Pflugers Arch. 463, 53–71. 10.1007/s00424-011-1014-6 21912835

[B14] Cudeiro J. Rivadulla C. Grieve K. L. (2000). A possible role for nitric oxide at the sleep/wake interface. Sleep 23, 829–835. 10.1093/sleep/23.6.1j 11007450

[B15] Destexhe A. Neubig M. Ulrich D. Huguenard J. (1998). Dendritic low-threshold calcium currents in thalamic relay cells. J. Neurosci. 18, 3574–3588. 10.1523/jneurosci.18-10-03574.1998 9570789 PMC6793130

[B16] Dixon D. B. Copenhagen D. R. (1997). Metabotropic glutamate receptor-mediated suppression of an inward rectifier current is linked via a cGMP cascade. J. Neurosci. 17, 8945–8954. 10.1523/jneurosci.17-23-08945.1997 9364042 PMC6573620

[B17] Fuentealba P. Steriade M. (2005). The reticular nucleus revisited: intrinsic and network properties of a thalamic pacemaker. Prog. Neurobiol. 75, 125–141. 10.1016/j.pneurobio.2005.01.002 15784303

[B18] Gabbott P. L. A. Bacon S. J. (1994). Two types of interneuron in the dorsal lateral geniculate nucleus of the rat: a combined NADPH diaphorase histochemical and GABA immunocytochemical study. J. Comp. Neurol. 350, 281–301. 10.1002/cne.903500211 7884043

[B19] Gibb B. J. Garthwaite J. (2001). Subunits of the nitric oxide receptor, soluble guanylyl cyclase, expressed in rat brain. Eur. J. Neurosci. 13, 539–544. 10.1046/j.1460-9568.2001.01421.x 11168561

[B20] Giuili G. Luzi A. Poyard M. Guellaën G. (1994). Expression of mouse brain soluble guanylyl cyclase and NO synthase during ontogeny. Brain Res. Dev. Brain Res. 81, 269–283. 10.1016/0165-3806(94)90313-1 7529143

[B21] Gonzalez-Forero D. Portillo F. Gomez L. Montero F. Kasparov S. Moreno-Lopez B. (2007). Inhibition of resting potassium conductances by long-term activation of the NO/cGMP/protein kinase G pathway: a new mechanism regulating neuronal excitability. J. Neurosci. 27, 6302–6312. 10.1523/jneurosci.1019-07.2007 17554004 PMC6672157

[B22] Hars B. (1999). Endogenous nitric oxide in the rat pons promotes sleep. Brain Res. 816, 209–219. 10.1016/s0006-8993(98)01183-4 9878741

[B23] He C. Chen F. Li B. Hu Z. (2014). Neurophysiology of HCN channels: from cellular functions to multiple regulations. Prog. Neurobiol. 112, 1–23. 10.1016/j.pneurobio.2013.10.001 24184323

[B24] Hess D. T. Matsumoto A. Kim S.-O. Marshall H. E. Stamler J. S. (2005). Protein S-nitrosylation: purview and parameters. Nat. Rev. Mol. Cell Biol. 6, 150–166. 10.1038/nrm1569 15688001

[B25] Hines M. L. Carnevale N. T. (2001). NEURON: a tool for neuroscientist. Neuroscientist 7, 123–135. 10.1177/107385840100700207 11496923

[B26] Hughes S. W. Cope D. W. Crunelli V. (1998). Dynamic clamp study of I_h_ modulation of burst firing and δ oscillations in thalamocortical neurons *in vitro*. Neuroscience 87, 541–550. 10.1016/s0306-4522(98)00170-5 9758221

[B27] Huguenard J. R. Prince D. A. (1994). Intrathalamic rhythmicity studied *in vitro*: nominal T-current modulation causes robust antioscillatory effects. J. Neurosci. 14, 5485–5502. 10.1523/jneurosci.14-09-05485.1994 8083749 PMC6577071

[B28] Kalinchuk A. V. Lu Y. Stenberg D. Rosenberg P. A. Porkka-Heiskanen T. (2006). Nitric oxide production in the basal forebrain is required for recovery sleep. J. Neurochem. 99, 483–498. 10.1111/j.1471-4159.2006.04077.x 17029601

[B29] Kanyshkova T. Meuth P. Bista P. Liu Z. Ehling P. Caputi L.. (2012). Differential regulation of HCN channel isoform expression in thalamic neurons of epileptic and non-epileptic rat strains. Neurobiol. Dis. 45, 450–461. 10.1016/j.nbd.2011.08.032 21945537 PMC3225716

[B30] Kanyshkova T. Pawlowski M. Meuth P. Dube C. Bender R. A. Brewster A. L.. (2009). Postnatal expression pattern of HCN channel isoforms in thalamic neurons: relationship to maturation of thalamocortical oscillations. J. Neurosci. 29, 8847–8857. 10.1523/jneurosci.0689-09.2009 19587292 PMC2768285

[B31] Kasten M. R. Rudy B. Anderson M. P. (2007). Differential regulation of action potential firing in adult murine thalamocortical neurons by Kv3.2, Kv1, and SK potassium and N-type calcium channels. J. Physiol. 584, 565–582. 10.1113/jphysiol.2007.141135 17761775 PMC2277158

[B32] Kim H. W. Park J.-S. Jeong H.-S. Jang M. J. Kim B.-C. Kim M.-K.. (2004). Nitric oxide modulation of the spontaneous firing of rat medial vestibular nuclear neurons. J. Pharmacol. Sci. 96, 224–228. 10.1254/jphs.scj04006x 15492461

[B33] Kostin A. McGinty D. Szymusiak R. Alam M. N. (2013). Sleep-wake and diurnal modulation of nitric oxide in the perifornical-lateral hypothalamic area: real-time detection in freely behaving rats. Neuroscience 254, 275–284. 10.1016/j.neuroscience.2013.09.022 24056193

[B34] Kuisle M. Wanaverbecq N. Brewster A. L. Frère S. G. A. Pinault D. Baram T. Z.. (2006). Functional stabilization of weakened thalamic pacemaker channel regulation in rat absence epilepsy. J. Physiol. 575, 83–100. 10.1113/jphysiol.2006.110486 16728450 PMC1819420

[B35] Leist M. Datunashvilli M. Kanyshkova T. Zobeiri M. Aissaoui A. Cerina M.. (2016). Two types of interneurons in the mouse lateral geniculate nucleus are characterized by different h-current density. Sci. Rep. 6:24904. 10.1038/srep24904 27121468 PMC4848471

[B36] Llinás R. R. Ribary U. Jeanmonod D. Kronberg E. Mitra P. P. (1999). Thalamocortical dysrhythmia: a neurological and neuropsychiatric syndrome characterized by magnetoencephalography. Proc. Natl. Acad. Sci. U S A 96, 15222–15227. 10.1073/pnas.96.26.15222 10611366 PMC24801

[B37] Ludwig A. Budde T. Stieber J. Moosmang S. Wahl C. Holthoff K.. (2003). Absence epilepsy and sinus dysrhythmia in mice lacking the pacemaker channel HCN2. EMBO J. 22, 216–224. 10.1093/emboj/cdg032 12514127 PMC140107

[B100] Ma X.-Y. Yu J.-M. Zhang S.-Z. Liu X.-Y. Wu B.-H. Wei X.-L. (2011). External Ba^2+^block of the two-pore domain potassiumchannel TREK-1 defines conformational transition in its selectivity filter. J. Biol. Chem. 286, 39813–39822. 10.1074/jbc.M111.264788 21965685 PMC3220548

[B39] McCormick D. A. Pape H. C. (1990). Noradrenergic and serotonergic modulation of a hyperpolarization-activated cation current in thalamic relay neurones. J. Physiol. 431, 319–342. 10.1113/jphysiol.1990.sp018332 1712844 PMC1181776

[B40] Mergia E. Friebe A. Dangel O. Russwurm M. Koesling D. (2006). Spare guanylyl cyclase NO receptors ensure high NO sensitivity in the vascular system. J. Clin. Invest. 116, 1731–1737. 10.1172/jci27657 16614755 PMC1435723

[B41] Meuth P. Meuth S. G. Jacobi D. Broicher T. Pape H.-C. Budde T. (2005). Get the rhythm: modeling neuronal activity. J. Undergrad. Neurosci. Educ. 4, A1–A11. 23493337 PMC3592624

[B42] Meuth S. G. Budde T. Kanyshkova T. Broicher T. Munsch T. Pape H.-C. (2003). Contribution of TWIK-related acid-sensitive K^+^ channel 1 (TASK1) and TASK3 channels to the control of activity modes in thalamocortical neurons. J. Neurosci. 23, 6460–6469. 10.1523/jneurosci.23-16-06460.2003 12878686 PMC6740633

[B43] Meuth S. G. Kanyshkova T. Meuth P. Landgraf P. Munsch T. Ludwig A.. (2006). Membrane resting potential of thalamocortical relay neurons is shaped by the interaction among TASK3 and HCN2 channels. J. Neurophysiol. 96, 1517–1529. 10.1152/jn.01212.2005 16760342

[B44] Neitz A. Mergia E. Imbrosci B. Petrasch-Parwez E. Eysel U. T. Koesling D.. (2014). Postsynaptic NO/cGMP increases NMDA receptor currents via hyperpolarization-activated cyclic nucleotide-gated channels in the hippocampus. Cereb. Cortex 24, 1923–1936. 10.1093/cercor/bht048 23448871

[B101] Nolan M. F. Malleret G. Dudman J. T. Buhl D. L. Santoro B. Gibbs E.. (2004). A behavioral role for dendritic integration: HCN1 channels constrain spatial memory and plasticity at inputs to distal dendrites of CA1 pyramidal neurons. Cell 119, 719–732. 10.1016/j.cell.2004.11.020 15550252

[B45] Notomi T. Shigemoto R. (2004). Immunohistochemical localization of Ih channel subunits, HCN1–4, in the rat brain. J. Comp. Neurol. 471, 241–276. 10.1002/cne.11039 14991560

[B46] Oostenveld R. Fries P. Maris E. Schoffelen J.-M. (2011). FieldTrip: open source software for advanced analysis of MEG, EEG, and invasive electrophysiological data. Comput. Intell. Neurosci. 2011, 1–9. 10.1155/2011/156869 21253357 PMC3021840

[B47] Pape H. C. (1996). Queer current and pacemaker: the hyperpolarization-activated cation current in neurons. Annu. Rev. Physiol. 58, 299–327. 10.1146/annurev.ph.58.030196.001503 8815797

[B48] Pape H. C. Mager R. (1992). Nitric oxide controls oscillatory activity in thalamocortical neurons. Neuron 9, 441–448. 10.1016/0896-6273(92)90182-d 1326294

[B49] Russwurm M. Russwurm C. Koesling D. Mergia E. (2013). NO/cGMP: the past, the present and the future. Methods Mol. Biol. 1020, 1–16. 10.1007/978-1-62703-459-3_1 23709023

[B50] Steriade M. McCormick D. A. Sejnowski T. J. (1993). Thalamocortical oscillations in the sleeping and aroused brain. Science 262, 679–685. 10.1126/science.8235588 8235588

[B51] Thomzig A. Laube G. Prüss H. Veh R. W. (2005). Pore-forming subunits of K-ATP channels, Kir6.1 and Kir6.2, display prominent differences in regional and cellular distribution in the rat brain. J. Comp. Neurol. 484, 313–330. 10.1002/cne.20469 15739238

[B52] Thoonen R. Cauwels A. Decaluwe K. Geschka S. Tainsh R. E. Delanghe J.. (2015). Cardiovascular and pharmacological implications of haem-deficient NO-unresponsive soluble guanylate cyclase knock-in mice. Nat. Commun. 6:8482. 10.1038/ncomms9482 26442659 PMC4699393

[B53] Timofeev I. (2011). Neuronal plasticity and thalamocortical sleep and waking oscillations. Prog. Brain Res. 193, 121–144. 10.1016/B978-0-444-53839-0.00009-0 21854960 PMC3250382

[B54] Toyoda H. Saito M. Okazawa M. Hirao K. Sato H. Abe H.. (2010). Protein kinase G dynamically modulates TASK1-mediated leak K^+^ currents in cholinergic neurons of the basal forebrain. J. Neurosci. 30, 5677–5689. 10.1523/jneurosci.5407-09.2010 20410120 PMC6632358

[B55] Vincent S. R. (2000). The ascending reticular activating system - from aminergic neurons to nitric oxide. J. Chem. Neuroanat. 18, 23–30. 10.1016/s0891-0618(99)00048-4 10708916

[B57] Yang S. Cox C. L. (2008). Excitatory and anti-oscillatory actions of nitric oxide in thalamus. J. Physiol. 586, 3617–3628. 10.1113/jphysiol.2008.153312 18535092 PMC2538827

[B58] Yao W. D. Gainetdinov R. R. Arbuckle M. I. Sotnikova T. D. Cyr M. Beaulieu J. M.. (2004). Identification of PSD-95 as a regulator of dopamine-mediated synaptic and behavioral plasticity. Neuron 41, 625–638. 10.1016/s0896-6273(04)00048-0 14980210

[B59] Yue B. W. Huguenard J. R. (2001). The role of H-current in regulating strength and frequency of thalamic network oscillations. Thalamus Relat. Syst. 1, 95–103. 10.1016/S1472-9288(01)00009-7 18239728 PMC2222919

[B60] Zagotta W. N. Olivier N. B. Black K. D. Young E. C. Olson R. Gouaux E. (2003). Structural basis for modulation and agonist specificity of HCN pacemaker channels. Nature 425, 200–205. 10.3410/f.1006733.197501 12968185

[B61] Zobeiri M. Chaudhary R. Datunashvili M. Heuermann R. J. Lüttjohann A. Narayanan V.. (2018). Modulation of thalamocortical oscillations by TRIP8b, an auxiliary subunit for HCN channels. Brain Struct. Funct. 223, 1537–1564. 10.1007/s00429-017-1559-z 29168010 PMC5869905

[B62] Zong X. Krause S. Chen C.-C. Krüger J. Gruner C. Cao-Ehlker X.. (2012). Regulation of hyperpolarization-activated cyclic nucleotide-gated (HCN) channel activity by cCMP. J. Biol. Chem. 287, 26506–26512. 10.1074/jbc.M112.357129 22715094 PMC3410992

